# Autoimmune disease in offspring of mothers with metabolic dysfunction-associated steatotic liver disease (MASLD): a nationwide cohort study

**DOI:** 10.1038/s41598-026-46246-x

**Published:** 2026-04-09

**Authors:** Carole A. Marxer, Fahim Ebrahimi, David Bergman, Jiangwei Sun, Hannes Hagström, Marcus Thuresson, Olof Stephansson, Jonas F. Ludvigsson

**Affiliations:** 1 Department of Medical Epidemiology and Biostatistics, Karolinska Institutet, Stockholm, SE-171 77 Sweden; 2 Department of Gastroenterology and Hepatology, University Digestive Health Care Center Basel – Clarunis, Basel, Switzerland; 3 Division of Hepatology, Department of Upper GI, Karolinska University Hospital, Stockholm, Sweden; 4 Department of Medicine, Karolinska Institutet, Huddinge, Stockholm, Sweden; 5 Statisticon AB, Uppsala, Sweden; 6 Department of Medicine, Karolinska Institutet, Solna, Stockholm, Sweden; 7 Department of Women’s Health, Division of Obstetrics, Karolinska University Hospital, Stockholm, Sweden; 8 Department of Paediatrics, Örebro University Hospital, Örebro, Swedena Sweden; 9 Department of Medicine, Columbia University College of Physicians and Surgeons, New York, NY USA

**Keywords:** MASLD, NAFLD, Autoimmune disease, Pregnancy, Epidemiology, Diseases, Gastroenterology, Immunology, Medical research, Risk factors

## Abstract

Metabolic dysfunction-associated steatotic liver disease (MASLD) in pregnancy may disrupt physiological maternal immune adaptations, thereby altering fetal immune programming and increasing the long-term risk of autoimmune diseases (ADs) in offspring. This is a pressing public health concern, as both maternal MASLD and childhood-onset ADs are rising in prevalence worldwide. This nationwide cohort study included all singleton live-born offspring of mothers with biopsy-proven MASLD diagnosed in Sweden between 1992 and 2017. We matched the 239 identified MASLD offspring with up to 5 reference offspring (*n* = 1,131) of mothers without known MASLD by maternal age at delivery, calendar year of delivery, and parity. We used multivariable Cox proportional hazard models to estimate adjusted hazard ratios (aHRs) for any AD up until 2023. We performed stratified analyses by histological stage of maternal MASLD (simple steatosis versus severe MASLD defined as either steatohepatitis, any stage of liver fibrosis, or cirrhosis). During a median of 18.4 years of follow-up (interquartile range [IQR] 13.2–23.8), 15 exposed offspring (incidence rate [IR] 3.4/1000 person-years) vs. 40 reference offspring (IR 1.9/1000 person-years) were diagnosed with AD. This corresponded to an aHR of 1.20 (95% CI 0.57–2.53). The risk of AD remained unchanged among 175 offspring born to mothers with simple steatosis (aHR 0.84, 95% CI 0.29–2.45), and was higher, but not significant, in 64 offspring of mothers with severe MASLD (aHR 1.98, 95% CI 0.67–5.84). We did not identify an association of maternal MASLD with AD in offspring. Larger studies with follow-up extending beyond early adulthood are, however, needed.

**Supplementary Information:**

The online version contains supplementary material available at 10.1038/s41598-026-46246-x.

## Introduction

Metabolic dysfunction-associated steatotic liver disease (MASLD), previously known as non-alcoholic fatty liver disease (NAFLD), is a leading cause of chronic liver disease^1,2^. Its prevalence is increasing rapidly, particularly among young adults^3,4^. Notably, ≥ 10% of women of childbearing age are affected^5,6^, a trend driven in part by rising rates of obesity and type 2 diabetes^1,7^. Simple steatosis, the mildest histological manifestation of MASLD, can progress to more severe liver conditions such as metabolic dysfunction-associated steatohepatitis (MASH), varying stages of fibrosis, and ultimately cirrhosis.

Evidence on long-term health outcomes among offspring exposed *in utero* to maternal MASLD remains scarce^1,8–10^. Thus, the American^2^ and European^11^ practice guidelines do not issue specific recommendations on offspring monitoring.

MASLD during pregnancy is characterized by systemic inflammation and immune dysregulation^12^. Successful pregnancy depends on tightly controlled immune and inflammatory regulation to support implantation, placentation, and fetal development^13–15^. Disruption of these physiologic immune adaptations (e.g., by maternal metabolic–inflammatory stress) may alter fetal immune programming and thereby influence long-term immune function in offspring^16^. Because maternal immune regulation during pregnancy overlaps with immune pathways involved in autoimmunity, MASLD represents a plausible gestational exposure for investigation in relation to offspring autoimmune disease risk, a heterogeneous group including e.g., type 1 diabetes and psoriasis^17,18^.

We aimed to perform a matched cohort study by leveraging the Swedish nationwide ESPRESSO (*Epidemiology Strengthened by histoPathology Reports in Sweden*) cohort to study the risk of AD in offspring exposed *in utero* to maternal biopsy-proven MASLD compared to offspring of mothers without known MASLD.

## Methods

### Study design and data sources

We performed a population-based matched cohort study in singleton live-born offspring of mothers with biopsy-confirmed MASLD and matched reference offspring of mothers without known MASLD using the same study design as in our previous studies^8,10^.

We used data from the Swedish nationwide ESPRESSO cohort (1992–2023)^19^, containing liver biopsy information from all pathology departments in Sweden (*n* = 28). ESPRESSO is linked to nationwide Swedish healthcare registers by the unique personal identify number assigned to all Swedish residents^19–21^.

### Study population

**Fig. 1:**
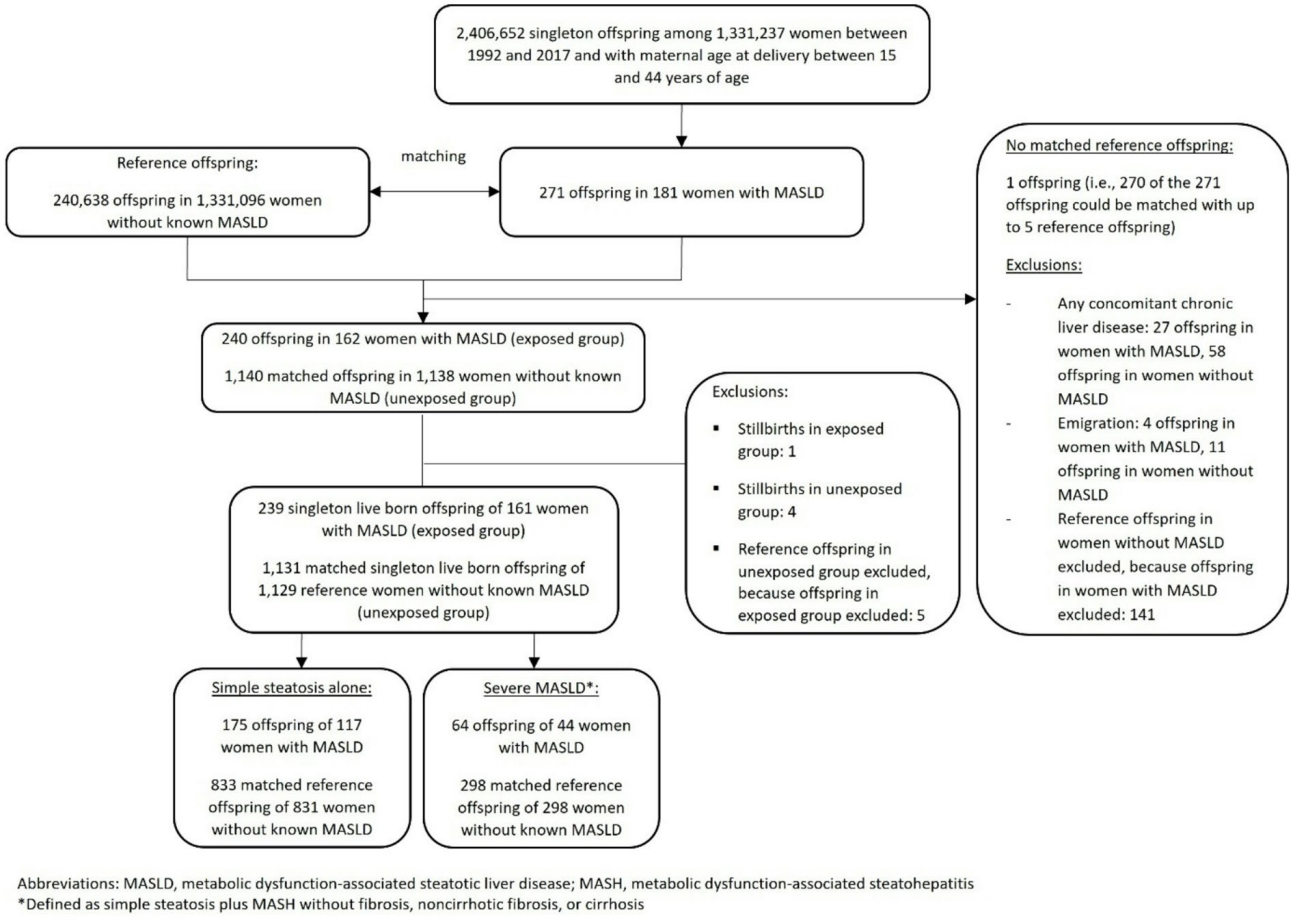
Flow chart of cohort enrolment. Notably, this flow chart has been presented in our earlier research^8,10^.

Our study population was constructed as follows: First, we identified singleton offspring born to mothers with biopsy-proven MASLD (definition below) between 15 and 44 years of age at delivery and between 1992 and 2017 in the *Medical Birth Register* (*MBR).*^22^ Second, we identified singleton offspring born to mothers without known MASLD (definition below) of the same age range at delivery and in the same time period (i.e., reference offspring). Third, we matched each singleton offspring of a mother with MASLD with ≤ 5 singleton offspring of reference mothers by maternal age at delivery, calendar year of delivery, and parity (Fig. 1). This base population has been described in detail in our earlier work on adverse pregnancy outcomes, including stillbirth^23^. In this study (and earlier work on offspring outcomes^8,10^, we further excluded stillbirth to focus on live-births. (Table S1). Notably, a woman could contribute several offspring to the study population.

To perform a cousin-controlled analysis, we identified all singleton offspring born to full sisters of the mothers with MASLD, but who did not have an *International Classification of Diseases* (ICD) code for MASLD (K76.0) as reference offspring (i.e., first cousins).

### Exposure

*In utero* exposure to maternal MASLD as defined in our previous studies:^8,10,23^ We required that women had a liver biopsy (topography T56) with a SNOMED morphology code for steatosis (M5008x or M5520x) between 1965 and 2017 (index liver biopsy). Among those, we required ≥ 1 subsequent pregnancy resulting in a delivery of an offspring. Specificity of our identification of MASLD was enhanced by using a validated ICD-based algorithm that follows international expert panel consensus recommendations^24^ resulting in a positive predictive value (PPV) of 92%^25^. This algorithm excluded patients with a recorded ICD-code for other concomitant chronic liver conditions (e.g., alcohol abuse; Table S2)^24^. According to this registry-based definition of MASLD, > 99.5% of patients with biopsy-confirmed NAFLD (i.e., previous terminology) meet the new MASLD criteria^26^.

To identify reference mothers without known MASLD and without other liver diseases (i.e., no *in utero* exposure to maternal MASLD), we followed the approach of our previous studies:^8,10,23^ We identified pregnant women without an ICD-code for MASLD prior to the date of delivery. Thereafter we excluded reference mothers with an ICD-code for other concomitant chronic liver conditions (Table S2) prior to the date of delivery, in accordance with the same exclusion criteria applied in the exposure group.

### Histological subgroups of MASLD

We used SNOMED definitions to determine histological subgroups of maternal MASLD^27^, and considered the following severity subgroups: simple steatosis, MASH without fibrosis, MASLD with noncirrhotic fibrosis, or cirrhosis due to MASLD (Table S3)^25^. Histological disease severity subgroups were based on the most recent histopathology report with MASLD before the date of delivery^28^. To enhance statistical power–given that individual severe histological subgroups were small–we pooled offspring of mothers with MASH with or without fibrosis and cirrhosis (“severe MASLD”) in stratified analyses by maternal MASLD severity.

### Outcome

We studied incident AD in offspring through 31 December 2023, defined as ≥ 1 primary or contributing ICD-code indicating AD (Table S4) recorded in the *National Patient Register* (*NPR*)^29^. We included 22 ADs as in another ESPRESSO study^30^(Addison’s disease, alopecia areata, ankylosing spondylitis, autoimmune hepatitis, autoimmune thyroiditis, celiac disease, dermatomyositis or juvenile dermatomyositis, grave’s disease, inflammatory bowel disease [IBD], multiple sclerosis, myasthenia gravis, polymyositis, primary biliary cholangitis, psoriasis, rheumatoid arthritis, sarcoidosis, Sjögren’s syndrome, spondyloarthritis, systemic sclerosis, systemic/cutaneous lupus erythematosus, type 1 diabetes, and vitiligo; Table S4). In a sensitivity analysis, AD was defined as ≥ 2 primary or contributing ICD-codes. Another sensitivity analysis defined AD as ≥ 1 ICD-code indicating AD or ≥ 1 prescription for the treatment of autoimmune thyroiditis (levothyroxine or liothyronine) or psoriasis, increasing the chances to capture autoimmune thyroiditis and psoriasis managed in primary care (i.e., not recorded in the *NPR*^29^, Table S4). Medications were identified through the *Prescribed Drug Register* (*PDR*; defined by *Anatomical Therapeutic Chemical* codes: Table S4)^31^.

### Follow-up

We followed offspring from one day after the offspring’s date of birth (index date) to prevent unintentional inclusion of stillborn offspring. Follow-up ended at the earliest of the following censoring reasons: (1) emigration from Sweden, (2) death, (3) 31 December 2023 (administrative censoring), or (4) outcome occurrence (Figure S1).

### Baseline characteristics and covariates

Maternal body mass index (BMI) and smoking status in early pregnancy were obtained from the *MBR*^22^.

We used the *NPR*^29^ and the *PDR*^31^ to retrieve data on additional maternal comorbidities and conditions, including autoimmune disease, diabetes, hypertension, dyslipidemia, and pre-eclampsia (Table S5)^29,31^. The *NPR* contains diagnoses and procedures from the inpatient setting since 1964, and diagnoses recorded during non-primary care outpatient visits since 2001^29^. The *PDR* contains information on medications dispensed at Swedish pharmacies since 2005, which was in addition to the *NPR* used to define certain maternal comorbidities and the outcome^31^.

We used the Swedish *Longitudinal Integrated Database for Health Insurance and Labour Market Studies* (*LISA*) to define the maternal education level^32^.

### Statistical analysis

We calculated the incidence rate (IR) per 1,000 person-years for AD during follow-up separately in offspring of mothers with MASLD and reference offspring. We used Cox proportional hazards regression to estimate crude and multivariable-adjusted hazard ratios (HRs) with 95% confidence intervals (CIs) for AD. Crude HRs (Model 1) were conditioned on the matching. Adjusted HRs (Model 2) were conditioned on the matching set and adjusted for five covariates including offspring sex and maternal factors including education level, any metabolic disorder recorded any time before delivery (any diabetes, any hypertension, obesity [early-pregnancy BMI ≥ 30 kg/m^2^], dyslipidemia, or pre-eclampsia), any AD except type 1 diabetes (already included in ‘any diabetes’) recorded any time before delivery, and early-pregnancy smoking status (Table S5).

We used robust standard errors to account for the correlation between offspring of the same mother.

We plotted crude cumulative incidence curves for AD by exposure status using the Aalen-Johansen estimator^33^.

In the primary analysis, we compared MASLD-exposed offspring with (unexposed) reference offspring regarding AD risk.

In a cousin-controlled analysis, we compared offspring of mothers with MASLD with offspring of their full sisters who did not have a diagnosis of MASLD (i.e., comparison of first cousins).

In pre-specified subgroup analyses, we determined the AD risk by histological subgroups of maternal MASLD (simple steatosis vs. severe MASLD), maternal age at delivery (< 35 vs. ≥35 years), maternal obesity in early pregnancy (< 30 vs. ≥30 kg/m^2^), presence of any maternal metabolic condition any time prior to delivery (obesity, diabetes, hypertension, dyslipidemia or pre-eclampsia), parity, (nulliparous vs. multiparous), offspring sex, and presence of maternal AD.

Pre-specified sensitivity analyses: (i) We restricted our population to offspring with normal birth weight for gestational age (i.e., not small for gestational age [SGA]), term-born (≥ 37 gestational weeks), and not delivered via cesarean section to determine if those parameters might mediate an association. (ii) We restricted our population to offspring of mothers with the index liver biopsy (i.e., first biopsy indicating MASLD) occurring before pregnancy and without an ICD-code for intrahepatic cholestasis of pregnancy (ICP; O26.6). Excluding offspring of mothers with the index liver biopsy during pregnancy minimizes the risk of including mothers with acute liver disease instead of MASLD. Excluding mothers with ICP allows us to determine if ICP contributes to the association under study. (iii) We assessed the AD risk using alternative outcome definitions.

Statistical analyses were performed in R version 4.3.1 (R Foundation for Statistical Computing, Vienna, Austria).

### Guidelines and regulations

All methods were performed in accordance with the relevant guidelines and regulations. First, this includes the implementation of the STROBE (STrengthening the Reporting of OBservational studies in Epidemiology) guidelines. Second, the Ethics Review Board in Stockholm (Sweden) approved the study (2014/1287-31/4, 2018/972 − 32 and 2022-05774-02) and waived informed consent.

## Results

### Study population

Our study population consisted of 239 live-born offspring of mothers with biopsy-proven MASLD and 1,131 matched reference offspring of mothers without known MASLD (Fig. 1). The proportion of female offspring (46%) was similar in both groups. Offspring of mothers with MASLD were > 3 times more likely to be born preterm (16.7% vs. 4.6%), twice as likely to be delivered via cesarean Sect.  (32.2% vs. 16.0%), and nearly twice as likely to be born SGA (14.7% vs. 8.6%) compared to reference offspring (Table 1). In both groups, the mothers were 32 years old at delivery (median; interquartile range [IQR] 27–36). Mothers with MASLD were more often smokers, had a lower level of education, more metabolic disorders, and were six times more likely to have an AD diagnosed prior to delivery of their offspring compared to reference mothers (18.4% vs. 3.0%; Table 1).

**Table 1:** Baseline characteristics of offspring born to mothers with MASLD and matched reference offspring of mothers without known MASLD.

	Offspring of mothers with MASLD	Reference offspring
**Offspring, n**	239	1131
**Unique mothers, n**	161	1129
**Years of follow-up**		
Median [IQR]	18.0 [13.4, 23.8]	18.5 [13.2, 23.8]
<10	31 (13.0)	157 (13.9)
10 to < 20	109 (45.6)	503 (44.5)
≥20	99 (41.4)	471 (41.6)
**Offspring characteristics**		
**Female sex**	108 (45.4)	528 (46.7)
**Calendar year of date of birth**		
1992–1999	64 (26.8)	308 (27.2)
2000–2010	125 (52.3)	595 (52.6)
2011–2017	50 (20.9)	228 (20.2)
**Gestational age at birth [days], median [IQR]**	274.0 [264.0, 283.0]	281.0 [274.0, 287.0]
**Preterm birth (< 37 weeks)**	40 (16.7)	52 (4.6)
**Fetal growth**		
**Birth weight [g]**		
Median [IQR]	3493 [2984, 3895]	3600 [3249, 3930]
Low (< 2,500)	26 (10.9)	38 (3.4)
Normal (2,500 to < 4,000)	166 (69.5)	853 (75.4)
High (≥ 4,000)	46 (19.2)	236 (20.9)
Missing	1 (0.4)	4 (0.4)
**Small for gestational age (SGA)**	35 (14.7)	97 (8.6)
**Cesarean section**	77 (32.2)	181 (16.0)
**Maternal characteristics**		
**Maternal age at delivery [years]**		
Median [IQR]	32.0 [27.0, 36.0]	32.0 [27.0, 36.0]
15 to < 25	26 (10.9)	123 (10.9)
25 to < 35	134 (56.1)	637 (56.3)
35 to 44	79 (33.1)	371 (32.8)
**Liver histology of maternal MASLD**		
Simple steatosis	175 (73.2)	-
MASH without fibrosis ^a^	30 (12.6)	-
Noncirrhotic fibrosis ^a^	30 (12.6)	-
Cirrhosis ^a^	4 (1.7)	-
**Year of first maternal MASLD diagnosis (index liver biopsy)**		
Up until 1999	161 (67.4)	-
2000–2010	71 (29.7)	-
2011–2017	7 (2.9)	-
**Disease duration (time between first MASLD diagnosis and delivery [years]**		
Median [IQR]	5.6 [3.1, 9.9]	-
<5	102 (42.7)	-
5 to < 10	80 (33.5)	-
≥10	57 (23.8)	-
**Maternal country of birth**		
Nordic	203 (84.9)	936 (82.8)
Other	36 (15.1)	195 (17.2)
**Civil status of the mother**		
Living with a partner	207 (86.6)	1018 (90.0)
Not living with a partner	11 (4.6)	21 (1.9)
Missing	21 (8.8)	92 (8.1)
**Education**		
Compulsory school (≤ 9 years)	36 (15.1)	116 (10.3)
Upper secondary school (10–12 years)	140 (58.6)	485 (42.9)
College or university (≥ 13 years)	63 (26.4)	508 (44.9)
Missing	0 (0.0)	22 (1.9)
**Parity: multiparous**	147 (61.5)	695 (61.5)
**BMI in early pregnancy [kg/m²]**		
Median [IQR]	28.7 [25.0, 33.2]	23.9 [21.5, 26.8]
<18.5	0 (0.0)	23 (2.0)
18.5 to < 25	55 (23.0)	585 (51.7)
25 to < 30	72 (30.1)	271 (24.0)
≥30	92 (38.5)	116 (10.3)
Missing	20 (8.4)	136 (12.0)
**Smoking in early pregnancy**		
Yes	41 (17.2)	114 (10.1)
No	187 (78.2)	959 (84.8)
Missing	11 (4.6)	58 (5.1)
**Prior comorbidities and conditions**		
Autoimmune disease	44 (18.4)	34 (3.0)
Diabetes ^b^	25 (10.5)	10 (0.9)
Hypertension ^c^	12 (5.0)	6 (0.5)
Dyslipidemia	4 (1.7)	2 (0.2)
Pre-eclampsia	19 (7.9)	35 (3.1)

Values are n (%), unless otherwise indicated.

MASLD, metabolic dysfunction-associated steatotic liver disease; n, number; IQR, interquartile range; MASH, metabolic dysfunction-associated steatohepatitis; BMI, body mass index.

^a^ In stratified analyses, MASH with or without fibrosis and cirrhosis were combined and categorized as severe MASLD.

^b^ Diabetes type 1, diabetes type 2, or gestational diabetes.

^c^ Including gestational hypertension.

Simple steatosis alone occurred in 73% of mothers (27% with severe MASLD; Table S6). AD was more common among mothers with simple steatosis (20.0% vs. 14.1% in severe MASLD).

Baseline characteristics are shown in Table 1/Table S6 and have been presented in our previous publication^8,10^.

### Any autoimmune disease

**Fig. 2:**
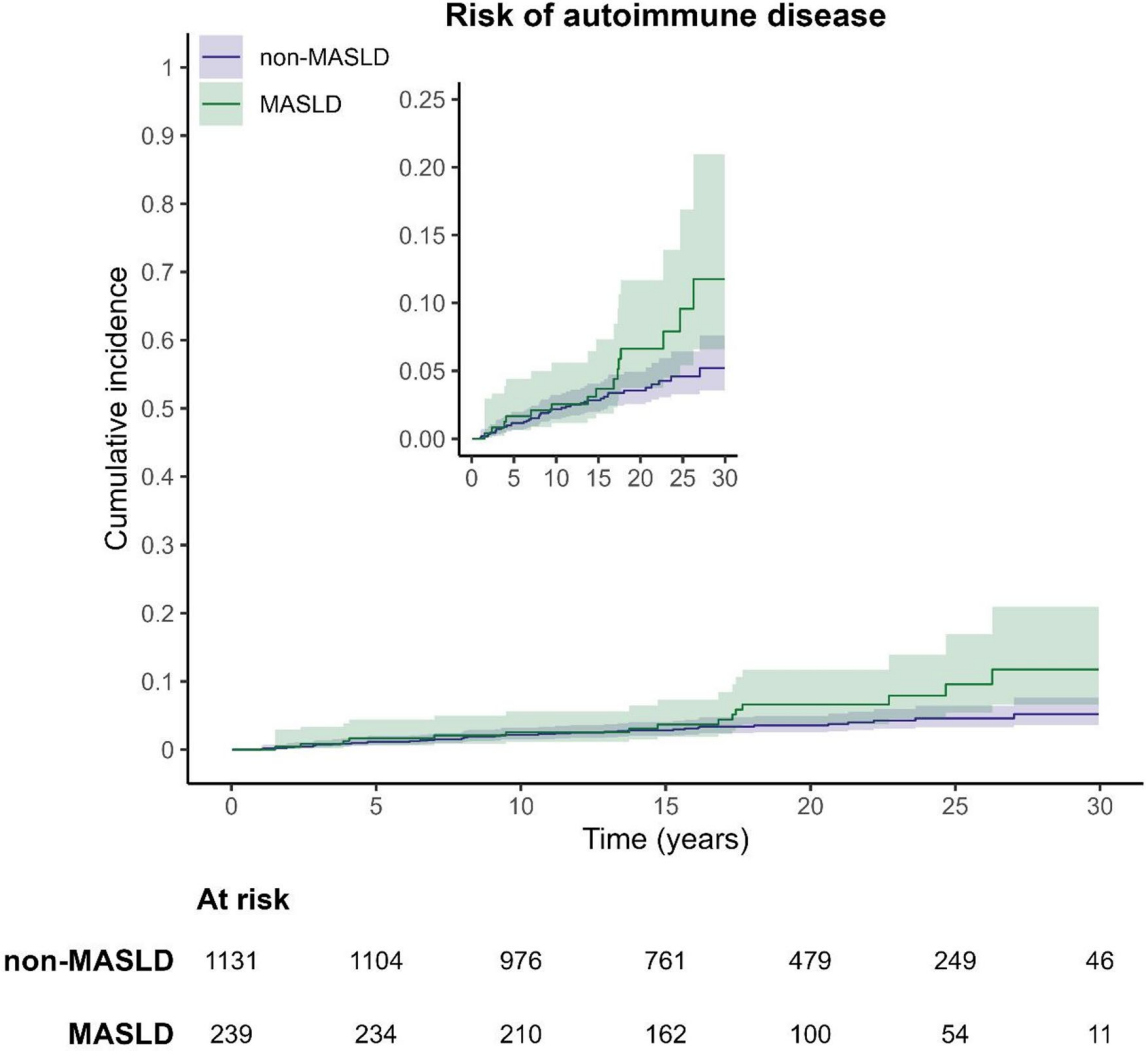
Crude cumulative incidence curves for autoimmune disease in offspring of mothers with MASLD versus reference offspring of mothers without known MASLD.

During a median follow-up of 18.0 years (IQR 13.4–23.8), 15 of 239 (6.3%) offspring of mothers with MASLD were diagnosed with an AD (IR 3.4 per 1000 person-years, 95% CI 1.9–5.6; Tables 2 and 3). During a similar follow-up in reference offspring (median: 18.5 years, IQR 13.2, 23.8), 40 of 1,131 (3.5%) reference offspring were diagnosed with an AD (IR 1.9 per 1000 person-years, 95% CI 1.4–2.6). The median age at AD diagnosis was higher among MASLD-exposed offspring (14.7 years, IQR 5.5–17.5 vs. 8.1 years, IQR 4.1–14.1 in reference offspring, Table 2). After multivariable adjustment, MASLD-exposed offspring had no increased hazards of AD compared to reference offspring (aHR 1.20, 95% CI 0.57–2.53, Table 3). Crude cumulative incidence curves diverged around early adulthood, with MASLD-exposed offspring more frequently diagnosed with AD after ~ 18 years of age (Fig. 2).

**Table 2:** Characteristics of autoimmune diseases.

	Overall	Offspring of mothers with MASLD	Reference offspring
**Number of offspring with autoimmune disease (%)**	55 (4.0)	15 (6.3)	40 (3.5)
**Age at autoimmune disease [years]**			
Median [IQR]	9.3 [4.1, 16.1]	14.7 [5.5, 17.5]	8.1 [4.1, 14.1]
<10	30 (54.5)	6 (40.0)	24 (60.0)
10 to < 20	17 (30.9)	6 (40.0)	11 (27.5)
≥20	8 (14.5)	3 (20.0)	5 (12.5)
**Type of autoimmune disease (in descending order** ^1^ **)**			
Celiac disease	15 (27.3)	2 (13.3)	13 (32.5)
Type 1 diabetes	14 (25.5)	3 (20.0)	11 (27.5)
Inflammatory bowel disease	7 (12.7)	4 (26.7)	3 (7.5)
Psoriasis	7 (12.7)	NR	NR
Alopecia areata	3 (5.5)	NR	NR
Grave’s disease	3 (5.5)	NR	NR
Spondyloarthritis	NR	NR	NR
Systemic/cutaneous lupus erythematosus	NR	NR	NR
Rheumatoid arthritis	NR	NR	NR
Vitiligo	NR	NR	NR

Values are n (%), unless otherwise indicated.

MASLD, metabolic dysfunction-associated steatotic liver disease; n, number; IQR, interquartile range, NR, not reported (data privacy concerns).

^1^ Table only shows specific autoimmune diseases for which two or more births with the diagnosis were found in both groups (protection of data privacy).

**Table 3:** Autoimmune disease among offspring exposed *in utero* to maternal MASLD and matched reference offspring of mothers without known MASLD.

	N	Events	py	IR per 1000 py (95% CI)	Crude HR (95% CI) Model 1*	Adjusted HR (95% CI) Model 2**
**Any autoimmune disease (main analysis)**						
Reference offspring	1131	40	20,777	1.9 (1.4–2.6)	1 (Reference)	1 (Reference)
Offspring of mothers with MASLD	239	15	4398	3.4 (1.9–5.6)	1.77 (0.98–3.20)	1.20 (0.57–2.53)
**Cousin-controlled analysis**						
Reference offspring	125	7	2569	2.7 (1.1–5.6)	1 (Reference)	1 (Reference)
Offspring of mothers with MASLD	78	5	1348	3.7 (1.2–8.7)	1.54 (0.49–4.88)	1.14 (0.28–4.63)
**Maternal disease severity**						
*Simple steatosis*						
Reference offspring	833	27	16,156	1.7 (1.1–2.4)	1 (Reference)	1 (Reference)
Offspring of mothers with MASLD	175	7	3422	2.0 (0.8–4.2)	1.22 (0.53–2.81)	0.84 (0.29–2.45)
**Severe MASLD°**						
Reference offspring	298	13	4620	2.8 (1.5–4.8)	1 (Reference)	1 (Reference)
Offspring of mothers with MASLD	64	8	976	8.2 (3.5–16.2)	2.86 (1.19–6.92)	1.98 (0.67–5.84)
**Maternal age at delivery**						
*<35 years*						
Reference offspring	760	28	14,752	1.9 (1.3–2.7)	1 (Reference)	1 (Reference)
Offspring of mothers with MASLD	160	12	3116	3.9 (2.0-6.7)	2.02 (1.03–3.97)	1.54 (0.70–3.40)
*≥35 years*						
Reference offspring	371	12	6024	2.0 (1.0-3.5)	1 (Reference)	1 (Reference)
Offspring of mothers with MASLD	79	3	1282	2.3 (0.5–6.8)	1.17 (0.33–4.15)	0.45 (0.09–2.20)
**Maternal BMI** **in early pregnancy**						
*<30 kg/m*^*2*^						
Reference offspring	624	22	11,866	1.9 (1.2–2.8)	1 (Reference)	1 (Reference)
Offspring of mothers with MASLD	147	10	2742	3.6 (1.7–6.7)	1.98 (0.94–4.19)	1.59 (0.70–3.61)
*≥30 kg/m*^*2*^						
Reference offspring	51	3	818	3.7 (0.8–10.7)	1 (Reference)	1 (Reference)
Offspring of mothers with MASLD	42	3	700	4.3 (0.9–12.5)	1.17 (0.24–5.78)	0.52 (0.03–9.09)
**≥ 1 Metabolic disorder any time prior to delivery°°**						
*Yes*	75	5	1166	4.3 (1.4–10.0)	1 (Reference)	1 (Reference)
Reference offspring	59	5	931	5.4 (1.7–12.5)	1.25 (0.36–4.31)	1.02 (0.16–6.42)
Offspring of mothers with MASLD						
*No*						
Reference offspring	478	19	9431	2.0 (1.2–3.1)	1 (Reference)	1 (Reference)
Offspring of mothers with MASLD	116	6	2250	2.7 (1.0-5.8)	1.33 (0.53–3.34)	1.40 (0.56–3.45)
**Parity**						
*Nulliparous*						
Reference offspring	436	10	7770	1.3 (0.6–2.4)	1 (Reference)	1 (Reference)
Offspring of mothers with MASLD	92	8	1630	4.9 (2.1–9.7)	3.80 (1.50–9.62)	2.45 (0.66–9.03)
*Multiparous*						
Reference offspring	695	30	13,007	2.3 (1.6–3.3)	1 (Reference)	1 (Reference)
Offspring of mothers with MASLD	147	7	2768	2.5 (1.0-5.2)	1.09 (0.48–2.49)	0.81 (0.31–2.16)
**Offspring sex**						
*Female*						
Reference offspring	242	9	4537	2.0 (0.9–3.8)	1 (Reference)	1 (Reference)
Offspring of mothers with MASLD	103	7	1889	3.7 (1.5–7.6)	1.94 (0.72–5.22)	1.77 (0.49–6.47)
*Male*						
Reference offspring	336	13	6121	2.1 (1.1–3.6)	1 (Reference)	1 (Reference)
Offspring of mothers with MASLD	127	7	2352	3.0 (1.2–6.1)	1.39 (0.56–3.49)	0.46 (0.12–1.81)
**Maternal autoimmune disease**						
*Yes*						
Reference offspring	8	NR	NR	NR	NR	NR
Offspring of mothers with MASLD	8	NR	NR	NR	NR	NR
*No*						
Reference offspring	900	33	17,052	1.9 (1.3–2.7)	1 (Reference)	1 (Reference)
Offspring of mothers with MASLD	195	12	3703	3.2 (1.7–5.7)	1.67 (0.86–3.23)	1.17 (0.55–2.51)

py, person-years; IR, incidence rate; CI, confidence interval; HR, hazard ratio; MASLD, metabolic dysfunction-associated steatotic liver disease; BMI, body mass index; NR, not reported (due to small sample size).

*Model 1: conditioned on matching set (maternal age at delivery, calendar year of delivery, parity).

**Model 2: conditioned on matching set and further adjusted for offspring sex and the following maternal factors: education level, any metabolic disorder recorded any time before delivery of the offspring of interest (any diabetes, any hypertension, obesity [BMI in early pregnancy ≥ 30 kg/m^2^], dyslipidemia, or pre-eclampsia), any autoimmune disease except type 1 diabetes recorded any time before delivery of the offspring of interest, and smoking status in early pregnancy.

### Specific autoimmune diseases

Among MASLD-exposed offspring, IBD was the most frequent AD (27% of all identified ADs vs. 8% among reference offspring), followed by type 1 diabetes (20%), and celiac disease (13%). Among reference offspring, celiac disease was most prominent (32%), followed by type 1 diabetes (28%), and psoriasis (15%, Table 2).

In a sensitivity analysis including medications in the definition of AD (i.e., antipsoriatics, levothyroxine, liothyronine), we identified a substantial number of additional events of psoriasis and autoimmune thyroiditis in both groups (Table S7): Psoriasis accounted for 23% of all ADs in MASLD-exposed offspring (vs. 15% in reference offspring), and autoimmune thyroiditis for 18% of all ADs in MASLD-exposed offspring (vs. 15% in reference offspring).

### Cousin-controlled analysis

This analysis included 78 offspring of mothers with MASLD (i.e., 33% of the study population) and 125 offspring of full sisters of the MASLD-mothers (i.e., first cousins), but who did not have a diagnosis of MASLD (Table S8). The aHR was nearly identical to that of the main analysis (1.14, 95% CI 0.28–4.63, Table 3).

### Histological subgroups of maternal MASLD

The hazard of AD was higher in offspring of mothers with severe MASLD (aHR 1.98, 95% CI 0.67–5.84) compared to those of mothers with simple steatosis (0.84, 95% CI 0.29–2.45) but did not reach statistical significance (Table 3).

### Subgroups

We did not find higher hazards of AD in any subgroup (Table 3), but statistical power was limited.

### Definitions of autoimmune disease

We did not observe an association between maternal MASLD and offspring AD when requiring a confirmatory AD diagnosis (aHR 0.71, 95% CI 0.28–1.78, Tables S9-10; Figure S2).

However, when AD was defined by either ≥ 1 diagnosis code or ≥ 1 medication, we observed a higher aHR compared to using ≥ 1 diagnosis code alone (1.65, 95% CI 0.88–3.11), though the association remained non-significant (Table S9). This was also reflected in the crude cumulative incidence curves (Figure S3), where the curves began to diverge even more clearly around early adulthood compared to the main analysis. More specifically, starting at ~ 18 years of age, MASLD-exposed offspring were more frequently diagnosed with AD than reference offspring.

### Sensitivity analyses

By restricting our population to offspring of mothers diagnosed with MASLD prior to pregnancy and without ICP, the aHR (95% CI) was nearly identical to those in the main analysis when (aHR 1.20, 95% CI 0.57–2.52, Table S9). Consistent results were also observed when restricting the analysis to offspring with normal birth weight for gestational age, born term, and not delivered via cesarean section (aHR 1.15, 95% CI 0.43–3.07, Table S9).

## Discussion

This nationwide study based on comprehensive Swedish registry data assessed for the first time the risk of AD among offspring exposed *in utero* to maternal MASLD. Our study included all 239 singleton live-born offspring of mothers with biopsy-confirmed MALSD in Sweden and 1,131 reference offspring of mothers without known MASLD. MASLD-exposure was not associated with statistically increased offspring AD, but a clinically meaningful risk cannot be ruled out.

This study contributes to the emerging picture of disease burden in offspring of mothers with MASLD^8–10^. Previously, we did not find evidence that maternal MASLD increases the risk of offspring mortality and cancer^8^, but we found a higher rate of serious infection^10^. Reassuringly, despite the potential for MASLD during pregnancy to disrupt physiological immune adaptations, as outlined in the introduction^16^, this cohort study found no association with AD through early adulthood. This finding that was consistent across subgroup and sensitivity analyses, but statistical power was limited.

However, MASLD-mothers themselves had more frequently an AD (18.4%) compared to reference mothers (3.0%). The higher frequency of MASLD in patients with AD has been described previously^34–38^, with medications to treat the underlying AD being a reason for more hepatic steatosis^35,36^. Previous studies have shown that maternal AD increases the risk of AD in the offspring, underscoring the importance of adjusting our analyses for maternal AD^39–41^. Interestingly, mothers with simple steatosis (i.e., mild histological stage of MASLD) more often had a concomitant AD compared to mothers with severe MASLD. It is possible that AD occurring in addition to severe MASLD may lead to reduced fertility, potentially reflecting a greater overall comorbidity burden. Another possible explanation is that these women may intentionally decide against pregnancy due to their comorbid condition.

Our study was possible given the long follow-up period (1992–2023) and almost no loss to follow-up, allowing us to assess the real-world risk of AD over time. While we did not observe an increased AD risk overall, the crude cumulative incidence curves showed more AD beyond ~ 18 years of age in MASLD-exposed offspring compared to reference offspring. The diverging crude cumulative incidence curves after 18 years of age is also reflected in the higher median age at first diagnosis of AD among MASLD-exposed offspring compared to reference offspring. There are several possible explanations. First, reference offspring may, on average, be leaner than MASLD-exposed offspring, in whom obesity may play a more significant role. Given that certain ADs are more likely to be investigated in individuals with underweight (particularly celiac disease and hyperthyroidism), it is possible that reference offspring are more frequently screened for AD during early childhood, leading to a higher number of diagnosed cases. Second, the onset of AD varies, with different types of AD developing at different ages^17^. It is possible that MASLD-exposed offspring may develop certain types of AD that typically emerge beyond or around age 18 (e.g., Graves’ disease, Hashimoto’s thyroiditis^17^, whereas reference offspring may develop ADs that tend to appear earlier in life. However, these explanations remain speculative, and the observed age difference at AD onset might be by chance.

Another strength of our study is the use of biopsy-confirmed MASLD, allowing us to stratify our analyses by MASLD histology. However, it was beyond the statistical power to rule out that the risk of AD increases with maternal MASLD severity. The risk estimates increased with maternal MASLD severity, but the AD risk remained non-significant among offspring of mothers with severe MASLD – either due to low power or due to a non-existent association.

As expected, the most frequent offspring AD were IBD, type 1 diabetes, and celiac disease, as well as psoriasis and autoimmune thyroiditis when AD medications were considered. We cannot rule out that *in utero* exposure to maternal MASLD is associated with an increased risk of certain specific ADs, as we were unable to analyze cause-specific risks due to limited statistical power, but high increases in risk seem unlikely given the modest HR for any autoimmunity (with an upper 95% CI of 2.53). Associations of *in utero* exposure to maternal MASLD and specific AD are however possible, as previous evidence suggests associations between MASLD and specific ADs in the same patient. For example, a large UK Biobank study suggests an increased risk of IBD among MASLD patients^42^ as postulated in previous studies^43,44^.

Preterm birth, cesarean section, and SGA were more frequent among MASLD-exposed offspring. Our previous study has shown that, even after adjusting for a wide range of key confounders, the risk of preterm birth and cesarean section were increased by 3.4- and 1.6-fold (no association observed for SGA) respectively^23^. In a sensitivity analysis, taking these pregnancy-related factors into account, we also did not observe an association between maternal MASLD and offspring AD either.

Similar to an earlier study, also using the Swedish *NPR* to ascertain AD, we defined AD disease based on ≥ 1 diagnosis code^30^. Also Conrad et al. used ≥ 1 diagnosis code for AD in their large cohort study on AD in 22 million individuals in the UK^17^. However, findings from a recent Swedish study^45^, which thoroughly evaluated AD definitions in Sweden’s registries, recently recommended requiring ≥ 2 AD recordings to confirm the disease. By applying this stricter AD definition, *in utero* exposure to maternal MASLD remained unassociated with offspring AD. The aHR decrease from 1.20 to 0.70 should be interpreted cautiously, as the first diagnosis of AD occurred later in MASLD-exposed offspring than in reference offspring, reducing the likelihood of a confirmatory diagnosis in MASLD-exposed offspring. Furthermore, the Swedish study^45^ indicates that the lack of primary care data in the Swedish *NPR*^29^ likely underestimates the prevalence of those ADs that are primarily diagnosed and treated in primary care (e.g., hypothyroidism and psoriasis; under review). We confirm this, as we identified more incident AD when considering treatments prescribed in primary care in the AD definition. Resulting adjusted risk estimates showed a slightly, but not statistically significant, increased risk of AD among MASLD-exposed offspring compared to reference offspring. Limited statistical power prevented definitive conclusions.

Our findings are reassuring for clinicians and women with MASLD and their children, but ultimate recommendations can only be provided after larger studies have been performed. For now, offspring do not seem to require specific monitoring for AD. However, physicians should be aware that AD may frequently co-exist with MASLD, adding another layer of complexity to their clinical management. Of note, our study does not imply that maternal MASLD has no impact on offspring in other regards. Current evidence on such risks remains extremely limited^8,10^. Our results might be implemented in the American^2^ and European^11^ clinical practice guidelines on MASLD during pregnancy, which do not yet provide evidence on long-term health among MASLD-exposed offspring.

Further strengths need to be considered. We used linked nationwide data from comprehensive Swedish healthcare registries to retrieve data on 99% of all births. This was possible due to the unique personal identity number enabling linkages of health records of an individual across registers, the possibility to link mother and infant’s health records, and the complete coverage of all registered individuals given the universal health care system. Further, data quality was high (e.g., PPV for most diagnoses in the *NPR* is 85–95%^19^). Further, we adjusted for an extensive set of confounders, including maternal BMI and smoking status which are reliably recorded in the *MBR* during the entire study period.^18^ Complete BMI is especially important given that maternal obesity is associated with a wide range of offspring outcomes^46^.

These limitations should be considered: We cannot rule out a slightly increased risk of AD, because undiagnosed MASLD among reference mothers may lead to an underestimation of AD risk (provided maternal MASLD is truly associated with offspring AD). On the other hand, MASLD-studies based on routinely collected data, such as ours, may overestimate the risk of clinical outcomes as individuals diagnosed with MASLD might be sicker than those with undiagnosed MASLD (who do not end up in our cohort), potentially leading to inflated risk estimates. While this is a limitation inherent to all MASLD-studies based on real-world data (i.e., no matter if ICD- or biopsy-based definitions of MASLD^25,47–49^, any potential overestimation in our study would represent even lower risk estimates reinforcing our conclusion that maternal MASLD does not seem to be associated with offspring AD. Further, multiple subgroup analyses may have meaningfully increased the risk of Type I errors. Therefore, we cannot rule out that specific subgroups of MASLD-exposed offspring may be linked to higher rates of offspring AD. Finally, our findings may not be generalizable to other ethnicities.

In conclusion, this nationwide study from Sweden found no association between maternal MASLD and AD among their offspring until early adulthood. However, a clinically meaningful risk of offspring AD cannot be ruled out. This initial evidence is nevertheless comforting to physicians and affected individuals. Studies using larger data sources with follow-up extending beyond early adulthood, and with more granular data on MASLD exposure, are however needed to extend and confirm our findings and to rule out a slight increase in the overall risk of AD and specific ADs.

## Supplementary Information

Below is the link to the electronic supplementary material.

Supplementary Material 1

## Data Availability

The datasets generated and/or analysed during the current study are not publicly available due Swedish data protection regulations but may be available from the corresponding author on reasonable request by complying with the General Data Protection Regulation, as well as national and institutional ethics regulations and standards.

## Abbreviations

AD: Autoimmune disease
aHR: Adjusted hazard ratio
ATC: Anatomical Therapeutic Chemical
BMI: Body mass index
CI: Confidence interval
ESPRESSO: Epidemiology Strengthened by histoPathology Reports in Sweden
ICD: International Classification of Diseases
IBD: Irritable bowel syndrome
ICP: Intrahepatic cholestasis of pregnancy
IQR: Interquartile range
IR: Incidence rate
LISA: Longitudinal Integrated Database for Health Insurance and Labour Market Studies
MASLD: Metabolic dysfunction-associated steatotic liver disease
MASH: Metabolic dysfunction-associated steatohepatitis
MBR: Medical Birth Register
NAFLD: Non-alcoholic fatty liver disease
NPR: National Patient Register
PDR: Prescribed Drug Register
PPV: Positive predictive value
py: Person-years
SGA: Small for gestational age

## References

[1] Sarkar, M. & Kushner, T. Metabolic dysfunction–associated steatotic liver disease and pregnancy. *J. Clin. Invest.* **135** (10). 10.1172/JCI186426 (May 2025). 10.1172/JCI186426 PMC12077888 40371643

[2] Sarkar, M. et al. Reproductive Health and Liver Disease: Practice Guidance by the American Association for the Study of Liver Diseases. *Hepatology* **73** (1), 318–365. 10.1002/hep.31559 (Jan. 2021). 10.1002/hep.31559 32946672

[3] Estes, C., Razavi, H., Loomba, R., Younossi, Z. & Sanyal, A. J. Modeling the epidemic of nonalcoholic fatty liver disease demonstrates an exponential increase in burden of disease. *Hepatology* **67** (1), 123–133. 10.1002/HEP.29466 (Jan. 2018). 10.1002/hep.29466 PMC5767767 28802062

[4] Allen, A. M. et al. Nonalcoholic fatty liver disease incidence and impact on metabolic burden and death: A 20 year-community study. *Hepatology* **67** (5), 1726–1736. 10.1002/HEP.29546 (May 2018). 10.1002/hep.29546 PMC5866219 28941364

[5] Vernon, G., Baranova, A. & Younossi, Z. M. Systematic review: the epidemiology and natural history of non-alcoholic fatty liver disease and non-alcoholic steatohepatitis in adults. *Aliment. Pharmacol. Ther.* **34** (3), 274–285. 10.1111/J.1365-2036.2011.04724.X (Aug. 2011). 10.1111/j.1365-2036.2011.04724.x 21623852

[6] Sarkar, M. et al. Sep., Non-alcoholic fatty liver disease in pregnancy is associated with adverse maternal and perinatal outcomes, *J. Hepatol.*, vol. 73, no. 3, pp. 516–522, (2020). 10.1016/J.JHEP.2020.03.049 10.1016/j.jhep.2020.03.049 PMC7438303 32531415

[7] Targher, G. et al. Prevalence of nonalcoholic fatty liver disease and its association with cardiovascular disease among type 2 diabetic patients. *Diabetes Care*. **30** (5), 1212–1218. 10.2337/DC06-2247 (May 2007). 10.2337/dc06-2247 17277038

[8] Marxer, C. A. et al. Mortality and Cancer in Offspring of Mothers With Biopsy-Proven MASLD During Pregnancy: A Nationwide Cohort Study. *Liver Int.* **45**, 70174. 10.1111/liv.70174 (2025). 10.1111/liv.70174 PMC12153413 40497679

[9] Marxer, C. A. & Ludvigsson, J. F. Response: Methodological Considerations for Assessing the Long-Term Impact of Maternal MASLD on Offspring Health. *Liver Int.* **45** (8). 10.1111/LIV.70233 (Aug. 2025). 10.1111/liv.70233 40682410

[10] Marxer, C. A. et al. Higher Risk of Serious Infection in Offspring of Mothers With Biopsy-Proven MASLD: A Nationwide Cohort Study. *United Eur. Gastroenterol. J.* **14** (1), e70163. 10.1002/UEG2.70163 (Feb. 2026). 10.1002/ueg2.70163 PMC12787312 41511818

[11] Williamson, C. et al. EASL Clinical Practice Guidelines on the management of liver diseases in pregnancy. *J. Hepatol.* **79** (3), 768–828. 10.1016/j.jhep.2023.03.006 (Sep. 2023). 10.1016/j.jhep.2023.03.006 37394016

[12] Jee, Y. M., Lee, J. Y. & Ryu, T. Chronic Inflammation and Immune Dysregulation in Metabolic-Dysfunction-Associated Steatotic Liver Disease Progression: From Steatosis to Hepatocellular Carcinoma. *Biomedicines 2025*. **13, Page 1260, 13**, (5), 1260. 10.3390/BIOMEDICINES13051260 (May 2025). 10.3390/biomedicines13051260 PMC12109540 40427086

[13] Denizli, M., Capitano, M. L. & Kua, K. L. Maternal obesity and the impact of associated early-life inflammation on long-term health of offspring. *Front. Cell. Infect. Microbiol.* **12**, 940937. 10.3389/FCIMB.2022.940937/PDF (Sep. 2022). 10.3389/fcimb.2022.940937 PMC9523142 36189369

[14] Mor, G., Aldo, P. & Alvero, A. B. The unique immunological and microbial aspects of pregnancy, *Nat. Rev. Immunol.*, vol. 17, no. 8, pp. 469–482, Aug. (2017). 10.1038/NRI.2017.64 10.1038/nri.2017.64 28627518

[15] Gude, N. M., Roberts, C. T., Kalionis, B. & King, R. G. Growth and function of the normal human placenta. *Thromb. Res.* **114**, 5–6. 10.1016/J.THROMRES.2004.06.038 (2004). 15507270 10.1016/j.thromres.2004.06.038

[16] Faas, M. M. & Smink, A. M. Shaping immunity: the influence of the maternal gut bacteria on fetal immune development, *Seminars in Immunopathology 2025 47:1*, vol. 47, no. 1, p. 13-, Feb. (2025). 10.1007/S00281-025-01039-8 10.1007/s00281-025-01039-8 PMC11787218 39891756

[17] Conrad, N. et al. Jun., Incidence, prevalence, and co-occurrence of autoimmune disorders over time and by age, sex, and socioeconomic status: a population-based cohort study of 22 million individuals in the UK, *The Lancet*, vol. 401, no. 10391, pp. 1878–1890, (2023). 10.1016/S0140-6736(23)00457-9 10.1016/S0140-6736(23)00457-9 37156255

[18] Xu, M., Wu, T., Li, Z. & Xin, G. Influence of genetically predicted autoimmune diseases on NAFLD. *Front. Immunol.* **14**10.3389/FIMMU.2023.1229570 (2023). 10.3389/fimmu.2023.1229570 PMC10520707 37767101

[19] Ludvigsson, J. F. & Lashkariani, M. Cohort profile: ESPRESSO (Epidemiology Strengthened by histoPathology Reports in Sweden). *Clin. Epidemiol.* **11**, 101–114. 10.2147/CLEP.S191914 (2019). 30679926 10.2147/CLEP.S191914 PMC6336132

[20] Ludvigsson, J. F., Otterblad-Olausson, P., Pettersson, B. U. & Ekbom, A. The Swedish personal identity number: possibilities and pitfalls in healthcare and medical research, *Eur. J. Epidemiol.*, vol. 24, no. 11, pp. 659–667, Nov. (2009). 10.1007/S10654-009-9350-Y 10.1007/s10654-009-9350-y PMC2773709 19504049

[21] Ludvigsson, J. F. & Lashkariani, M. Cohort Update: ESPRESSO (Epidemiology Strengthened by Histopathology Reports in Sweden). *Clin. Epidemiol.* **17**, 193–196. 10.2147/CLEP.S499859 (Feb. 2025). 10.2147/CLEP.S499859 PMC11871922 40027403

[22] Cnattingius, S. et al. The Swedish medical birth register during five decades: documentation of the content and quality of the register. *Eur. J. Epidemiol.* **38** (1), 109–120. 10.1007/S10654-022-00947-5 (Jan. 2023). 10.1007/s10654-022-00947-5 PMC9867659 36595114

[23] Marxer, C. A. et al. Adverse pregnancy and birth outcomes in women with biopsy-proven MASLD: a nationwide cohort study. *EClinicalMedicine* 103238. 10.1016/J.ECLINM.2025.103238 (May 2025). 10.1016/j.eclinm.2025.103238 PMC12235392 40630617

[24] Hagström, H. et al. Jul., Administrative Coding in Electronic Health Care Record-Based Research of NAFLD: An Expert Panel Consensus Statement, *Hepatology*, vol. 74, no. 1, pp. 474–482, (2021). 10.1002/HEP.31726 10.1002/hep.31726 PMC8515502 33486773

[25] Simon, T. G., Roelstraete, B., Khalili, H., Hagström, H. & Ludvigsson, J. F. Mortality in biopsy-confirmed nonalcoholic fatty liver disease: results from a nationwide cohort, *Gut*, vol. 70, no. 7, pp. 1375–1382, Jul. (2021). 10.1136/GUTJNL-2020-322786 10.1136/gutjnl-2020-322786 PMC8185553 33037056

[26] Hagström, H., Vessby, J., Ekstedt, M. & Shang, Y. 99% of patients with NAFLD meet MASLD criteria and natural history is therefore identical. *J. Hepatol.* **80** (2), e76–e77. 10.1016/J.JHEP.2023.08.026 (Feb. 2024). 10.1016/j.jhep.2023.08.026 37678723

[27] Svensk, F. & Patologi Svensk Förening för Klinisk Cytologi. Accessed: Apr. 04, [Online]. (2019). Available: https://www.svfp.se/foreningar/uploads/L15178/kvast/lever/Leverbiopsier2019.pdf

[28] McPherson, S. et al. Evidence of NAFLD progression from steatosis to fibrosing-steatohepatitis using paired biopsies: Implications for prognosis and clinical management. *J. Hepatol.* **62** (5), 1148–1155. 10.1016/j.jhep.2014.11.034 (May 2015). 10.1016/j.jhep.2014.11.034 25477264

[29] Ludvigsson, J. F. et al. External review and validation of the Swedish national inpatient register. *BMC Public. Health*. **11**, 450. 10.1186/1471-2458-11-450 (2011). 21658213 10.1186/1471-2458-11-450 PMC3142234

[30] Yuan, S. et al. Older age of celiac disease diagnosis and risk of autoimmune disease: A nationwide matched case-control study. *J. Autoimmun.* **143**10.1016/J.JAUT.2024.103170 (Feb. 2024). 10.1016/j.jaut.2024.103170 38286066

[31] Wettermark, B. et al. Jul., The new Swedish Prescribed Drug Register—Opportunities for pharmacoepidemiological research and experience from the first six months, *Pharmacoepidemiol. Drug Saf.*, vol. 16, no. 7, pp. 726–735, (2007). 10.1002/PDS.1294 10.1002/pds.1294 16897791

[32] Ludvigsson, J. F., Svedberg, P., Olén, O., Bruze, G. & Neovius, M. The longitudinal integrated database for health insurance and labour market studies (LISA) and its use in medical research. *Eur. J. Epidemiol.* **34** (4), 423–437. 10.1007/S10654-019-00511-8/FIGURES/6 (Mar. 2019). 10.1007/s10654-019-00511-8 PMC6451717 30929112

[33] Putter, H., Fiocco, M. & Gekus, R. B. Tutorial in biostatistics: competing risks and multi-state models. *Stat. Med.* **26** (11), 2389–2430. 10.1002/SIM.2712 (May 2007). 10.1002/sim.2712 17031868

[34] De Vries, M., Westerink, J., Kaasjager, K. H. A. H. & De Valk, H. W. Prevalence of Nonalcoholic Fatty Liver Disease (NAFLD) in patients with type 1 diabetes mellitus: A systematic review and meta-analysis. *J. Clin. Endocrinol. Metab.* **105** (12). 10.1210/CLINEM/DGAA575 (Dec. 2020). 10.1210/clinem/dgaa575 PMC7526735 32827432

[35] Zou, Z. Y., Shen, B. & Fan, J. G. Systematic Review with Meta-analysis: Epidemiology of Nonalcoholic Fatty Liver Disease in Patients with Inflammatory Bowel Disease, *Inflamm. Bowel Dis.*, vol. 25, no. 11, pp. 1764–1772, Oct. (2019). 10.1093/IBD/IZZ043. 10.1093/ibd/izz043 30918952

[36] Hoffmann, P., Jung, V., Gauss, A. & Behnisch, R. Prevalence and risk factors of nonalcoholic fatty liver disease in patients with inflammatory bowel diseases: A cross-sectional and longitudinal analysis, *World J. Gastroenterol.*, vol. 26, no. 46, pp. 7367–7381, Dec. (2020). 10.3748/WJG.V26.I46.7367. 10.3748/wjg.v26.i46.7367 PMC7739163 33362390

[37] Reilly, N. R., Lebwohl, B., Hultcrantz, R., Green, P. H. R. & Ludvigsson, J. F. Increased risk of non-alcoholic fatty liver disease after diagnosis of celiac disease, *J. Hepatol.*, vol. 62, no. 6, pp. 1405–1411, Jun. (2015). 10.1016/j.jhep.2015.01.013 10.1016/j.jhep.2015.01.013 PMC4439270 25617505

[38] Valvano, M. et al. Celiac disease, gluten-free diet, and metabolic and liver disorders, *Nutrients*, vol. 12, no. 4, Apr. (2020). 10.3390/NU12040940. 10.3390/nu12040940 PMC7230624 32231050

[39] Yen, F. S., Huang, J. Y., Lin, S. Y., Liao, P. L. & Wei, J. C. C. Maternal autoimmune disease associated with a higher risk of offspring with type 1 diabetes: A nationwide mother-child cohort study in Taiwan. *Diabetes Metab.* **49** (3). 10.1016/j.diabet.2023.101443 (May 2023). 10.1016/j.diabet.2023.101443 36972847

[40] Shao, Y. H. J. & Chen, Y. M. Parental autoimmunity and offspring risks of rheumatic diseases: a nationwide population-based study, *Rheumatology (Oxford).*, vol. 63, no. 8, pp. 2189–2198, Aug. (2024). 10.1093/RHEUMATOLOGY/KEAD562 10.1093/rheumatology/kead562 37878801

[41] Neuhausen, S. L. et al. Sep., Co-occurrence of celiac disease and other autoimmune diseases in celiacs and their first-degree relatives, *J. Autoimmun.*, vol. 31, no. 2, p. 160, (2008). 10.1016/J.JAUT.2008.06.001 10.1016/j.jaut.2008.06.001 PMC2630860 18692362

[42] Zhang, Q. et al. Apr., Long-Term Risk of Inflammatory Bowel Disease With MASLD: A Large-Scale Prospective Cohort Study in UK Biobank, *Journal of Gastroenterology and Hepatology (Australia)*, (2025). 10.1111/JGH.16880 10.1111/jgh.16880 39828371

[43] Zhang, Q. et al. Jul., Non-alcoholic fatty liver degree and long-term risk of incident inflammatory bowel disease: A large-scale prospective cohort study, *Chin. Med. J. (Engl).*, vol. 137, no. 14, pp. 1705–1714, (2024). 10.1097/CM9.0000000000002859. 10.1097/CM9.0000000000002859 PMC11268827 37962217

[44] Chen, J. et al. Metabolic dysfunction-associated fatty liver disease and liver function markers are associated with Crohn’s disease but not Ulcerative Colitis: a prospective cohort study. *Hepatol. Int.* **17** (1), 202–214. 10.1007/S12072-022-10424-6 (Feb. 2023). 10.1007/s12072-022-10424-6 PMC9895026 36194337

[45] Bergman, D. et al. Incidence and prevalence of autoimmune disease in the Swedish National Patient Register. *Eur. J. Epidemiol.*10.1007/s10654-026-01369-3 (Feb. 2026). 10.1007/s10654-026-01369-3 41721992

[46] Hagström, H. et al. Maternal obesity increases the risk and severity of NAFLD in offspring, *J. Hepatol.*, vol. 75, no. 5, pp. 1042–1048, Nov. (2021). 10.1016/j.jhep.2021.06.045 10.1016/j.jhep.2021.06.045 34289397

[47] Issa, G., Shang, Y., Strandberg, R., Hagström, H. & Wester, A. Cause-specific mortality in 13,099 patients with metabolic dysfunction-associated steatotic liver disease in Sweden. *J. Hepatol.* **0** (0). 10.1016/J.JHEP.2025.03.001 (Mar. 2025). 10.1016/j.jhep.2025.03.001 40139508

[48] Shang, Y. et al. Risk of infections in non-alcoholic fatty liver disease: A nationwide population-based cohort study, *Liver Int.*, vol. 43, no. 10, pp. 2142–2152, Oct. (2023). 10.1111/LIV.15680 10.1111/liv.15680 37475642

[49] Ebrahimi, F. et al. Dec., Risk of Severe Infection in Patients With Biopsy-proven Nonalcoholic Fatty Liver Disease - A Population-based Cohort Study, *Clin. Gastroenterol. Hepatol.*, vol. 21, no. 13, pp. 3346–3355.e19, (2023). 10.1016/J.CGH.2023.05.013 10.1016/j.cgh.2023.05.013 37245712

