# Supplementary material for: Autoimmune disease in offspring of mothers with metabolic dysfunction-associated steatotic liver disease (MASLD): a nationwide cohort study
Source: Sci Rep. 2026 Apr 9;16:12217. doi: 10.1038/s41598-026-46246-x (PMC13076799; doi:10.1038/s41598-026-46246-x)
Supplement: Supplementary file 1 — Supplementary Material 1 [file 41598_2026_46246_MOESM1_ESM.docx]

**SUPPLEMENTARY MATERIAL**

**Title:** Autoimmune disease in offspring of mothers with metabolic dysfunction-associated steatotic liver disease (MASLD): A nationwide cohort study

**Authors:** Carole A. Marxer, Fahim Ebrahimi, David Bergman, Jiangwei Sun, Hannes Hagström, Marcus Thuresson, Olof Stephansson, Jonas F. Ludvigsson

Contents

[**Figure S1:** Study design. 2](#_Toc223347843)

[**Table S1:** Exclusion of stillbirths. 2](#_Toc223347844)

[**Table S2:** Exclusion of any concomitant chronic liver condition among mothers with MASLD and reference mothers 3](#_Toc223347845)

[**Table S3:** Definitions of histological severity groups of maternal MASLD 4](#_Toc223347846)

[**Table S4:** Codes to identify autoimmune disease (i.e., outcome) in the offspring. 5](#_Toc223347847)

[**Table S5:** Definitions of covariates and baseline characteristic variables (as previously described^1–3^). 7](#_Toc223347848)

[**Table S6:** Baseline characteristics of I. offspring of mothers with simple steatosis alone versus reference offspring of mothers without MASLD, as well as baseline characteristics of II. offspring of mothers with severe MASLD versus matched reference offspring of mothers without MASLD. 9](#_Toc223347849)

[**Table S7:** Characteristics of autoimmune disease defined by ≥1 diagnosis code for autoimmune disease or ≥1 prescribed medication uniquely indicated for autoimmune disease during follow-up (sensitivity analysis). 13](#_Toc223347850)

[**Table S8:** Cousin-controlled analysis: Baseline characteristics of mothers with MASLD, their sisters (without known MASLD), and their offspring. 14](#_Toc223347851)

[**Table S9:** Sensitivity analyses: Autoimmune disease among offspring exposed in utero to maternal MASLD and reference offspring. 17](#_Toc223347852)

[**Table S10:** Characteristics of the outcome when autoimmune disease was confirmed by a second (confirmatory) diagnosis during follow-up. 18](#_Toc223347853)

[**Figure S2:** Crude cumulative incidence curves for confirmed autoimmune disease (≥2 diagnosis codes) in offspring born to mothers with MASLD vs. reference offspring of mothers without known MASLD. 19](#_Toc223347854)

[**Figure S3:** Crude cumulative incidence curves for autoimmune disease (≥1 diagnosis or ≥1 medication) in offspring born to mothers with MASLD vs. reference offspring of mothers without known MASLD. 20](#_Toc223347855)

[**References (Supplementary Material)** 21](#_Toc223347856)


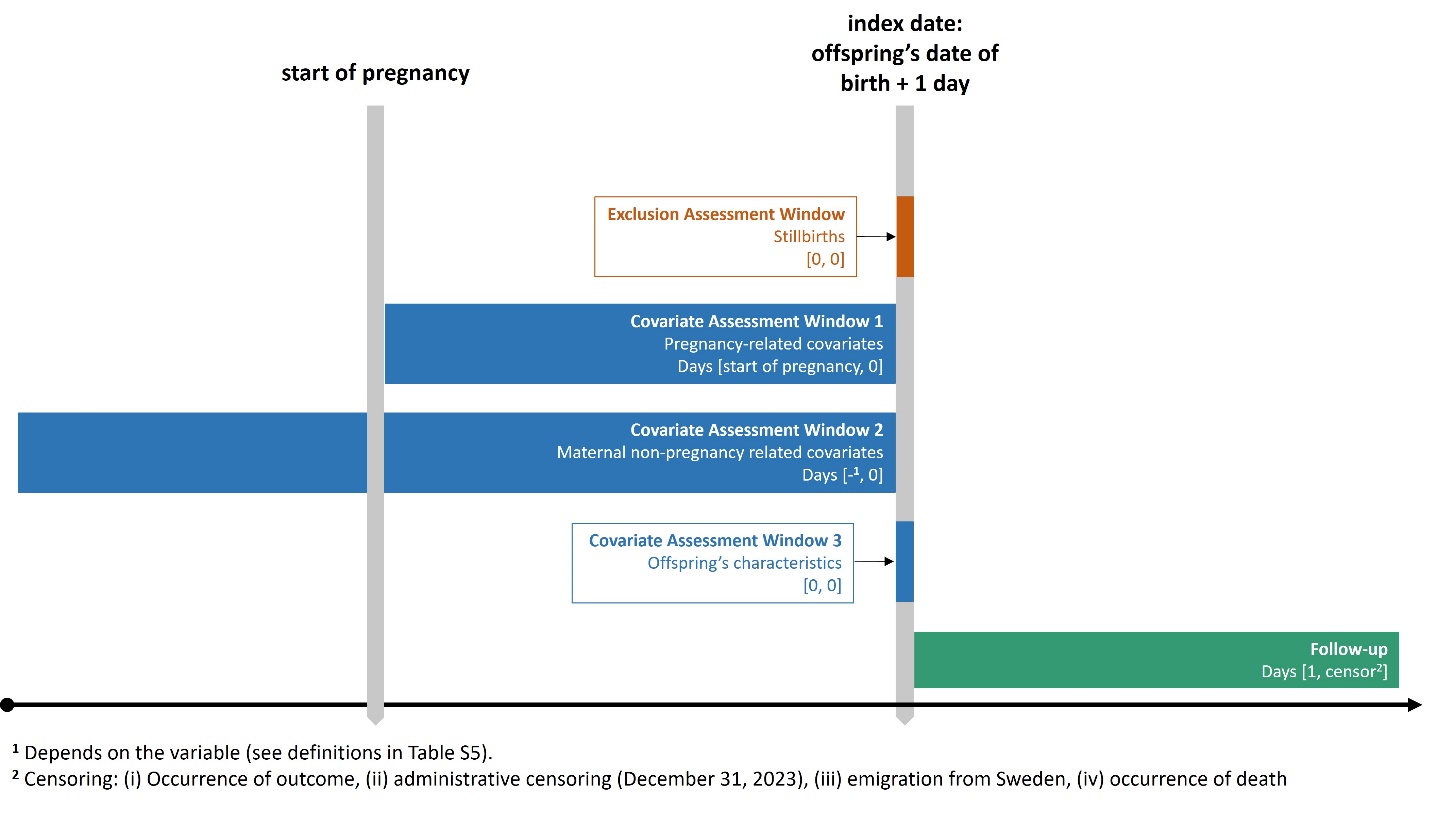


**Figure S1:** Study design. Figure adapted from Marxer et al.^1,2^

**Table S1:** Exclusion of stillbirths.

| Excluded condition | Definition | Data source |
| --- | --- | --- |
| Stillbirth | Death of fetus after 28 completed gestational weeks until July 2008 and thereafter after 22 completed gestational weeks. | *MBR* |

Abbreviations: *MBR, Medical Birth Register.*

**Table S2:** Exclusion of any concomitant chronic liver condition among mothers with MASLD and reference mothers (as previously described^1–3^).

| Excluded conditions^1^ | ICD-8 (1969-1986) | ICD-9 (1987-1996) | ICD-10 (1997-) |
| --- | --- | --- | --- |
| Alcohol abuse / misuse, or Alcohol-related liver disease | 280,00; 281,00; 307,00; 307,10; 307,99; 322; 581,10; 583,10; 261,00; 262,00; 291; 291,1; 303; 571,00; 571,01; 979; 980,00; 980,01; 980,98; 980,99 | 291; 294A; 303; 305A; 357F; 425F; 535D; 571A-D; 760W; 790D; 977D; 980A; 980X; V97B | E24.4; F10; G31.2; G62.1; G72.1; I42.6; K29.2; K70; K85.2; K86.0; Q35.4; R78.0; T51.0; T51.8; T51.9; X65; Y15; Y57.3; Y90; Y91; Z50.2; Z71.4; Z72.1 |
| Other abuse- and drug-related diagnoses | 571,0; E860; N980 | 571A-D | F11-F19 |
| Drug-induced liver disease | − | 573D | K71 |
| Viral hepatitis (e.g. hepatitis B, C) | 070; 999,20 | 070 | B15-19; B00.8; B25.1 |
| Budd-Chiari | − | 453A | I82 |
| Liver abscess | 572 | 572A | K75.0; A06.4 |
| HIV | 079,83; Y40,49; Y41,49 | 279K | B20-B24; F02.4; Z21.9; Z71.7 |
| Hemochromatosis | 273,2 | 275A | E83.1 |
| Wilson’s disease | 273,3 | 275B | E83.0 |
| Autoimmune hepatitis | − | 573D; 571E | K75.4 |
| Primary biliary cholangitis | − | 571G | K74.3; K74.4 |
| Other cholangitis | 574,06 | 576B | K83; K83.0A |
| Alpha-1 antitrypsin deficiency | − | 277G | E88.0 |
| Glycogen storage disease |  | 271W, 271X | E74 |
| Liver transplantation^2^ | − | V42H 5200-5299^†^ | Z94.4 JJC, DJ005; DJ006^†^ |
| Gastric bypass surgery | − | − | JDF^†^ |

Abbreviations: ICD, International Classification of Disease; HIV, human immunodeficiency virus

^1^ We will exclude any person with a diagnosis for another etiology of liver disease, or alcohol abuse/misuse or alcohol-related liver disease, defined on or prior to the index liver biopsy date.

^2^ Liver transplantation and bariatric surgery were further defined via procedure codes.

**Table S3:** Definitions of histological severity groups of maternal MASLD (as previously described^1–3^).

|  | | SNOMED and ICD codes | |
| --- | --- | --- | --- |
| # | Histological subgroup | Inclusion | Exclusion |
| 1 | **Cirrhosis** | M495 [exactly] or M4950x | - |
| 2 | **Noncirrhotic fibrosis (note that this may or may not include MASH)** | Steatosis: either M008x or M5520x, PLUS at least 1 fibrosis code: M49 [exactly], M4900x or M49060. | Cirrhosis codes: M459 [exactly] or M4950x |
| 3 | **MASH without fibrosis** | Steatosis: either M008x or M5520x, PLUS at least one of the following:  1. any M4- code, or 2. M5400x | M4 defines a very broad category of inflammation, both acute and chronic.  Cannot have any of:  Fibrosis codes (M49 [exactly], M4900x or M49060)  OR Cirrhosis: M495 [exactly] or M4950x. |
| 4 | **Simple steatosis** | M5008x or M5520x | Cannot have any:  Inflammation codes: M4- or M5400x  OR Fibrosis: M49 [exactly], M4900x or M49060  OR Cirrhosis: M495 [exactly] or M4950x |

Abbreviations: MASLD, metabolic dysfunction-associated steatotic liver disease; SNOMED, Systematized Nomenclature of Medicine; ICD, International Classification of Diseases.

Of note: In Sweden, clinically indicated liver biopsies are generally conducted with a single pass of the liver, unless a satisfactory specimen could not be obtained. According to Swedish liver histopathology reporting recommendations, it is documented if any biopsy is too short in length (i.e., <15 mm in length), has fewer than five portal tracts, or is fragmented, respectively.^4^

### **Table S4:** Codes to identify autoimmune disease (i.e., outcome) in the offspring.

| Autoimmune disease^1^ | ICD codes^2^ |
| --- | --- |
| Systemic/cutaneous lupus erythematosus | - ICD-8: 734,1 - ICD-9: 710A - ICD-10: M32, L931 |
| Vitiligo | - ICD-8: 709,05 - ICD-9: - - ICD-10: L80 |
| Psoriasis | - ICD-8: 696,0 and 696,1 - ICD-9: 696B - ICD-10: L40 - Sensitivity analysis: ATC: D05 (antipsoriatics) |
| Alopecia areata | - ICD-8: 704,00 - ICD-9: - - ICD-10: L63 |
| Systemic sclerosis | - ICD-8: 734,00-734,19 - ICD-9: 710B - ICD-10: M34 |
| Dermatomyositis or juvenile dermatomyositis | - ICD-8: 716,00 - ICD-9: 710D - ICD-10: M33 except M33.2 |
| Polymyositis | - ICD-8: 716,10 - ICD-9: 710E - ICD-10: M33.2 |
| Grave’s disease | - ICD-8: 242,0 - ICD-9: 242A - ICD-10: E05.0 |
| Type 1 diabetes | - ICD-8: 250 - ICD-9: 250 - ICD-10: E10   Of note, we do not include insulin in the sensitivity analysis, because type 1 diabetes will certainly be captured in outpatient/inpatient care (*National Patient Register*). Medications are only needed in sensitivity analysis to try to capture primary care diagnoses of autoimmune disease. |
| Autoimmune thyroiditis (Hashimoto's thyroiditis) | - ICD-8: 245,02 and 245,03 - ICD-9: 245C - ICD-10: E06.3 - Sensitivity analysis: ATC: H03AA01 (levothyroxine sodium), H03AA02 (liothyronine sodium) |
| Addison´s disease | - ICD-8: 255,10 - ICD-9: 255E - ICD-10: E27.1 and E27.2 |
| Autoimmune hepatitis | - ICD-8: 573,0 and 571,9 - ICD-9: 573D, 573E - ICD-10: K75.4 |
| Primary biliary cholangitis | - ICD-8: - - ICD-9: 571G - ICD-10: K74.3 |
| Inflammatory bowel disease | - ICD8: 569,04; 563,1; 563,10; 569,02; 563,00 - ICD-9: 555, 556 - ICD-10: K50, K51 and K52.3 |
| Sjögren syndrome | - ICD-8: 734,90 - ICD-9: 710C - ICD-10: M35.0 |
| Myasthenia gravis | - ICD-8: 733,00 - ICD-9: 358A - ICD-10: G70.0 |
| Rheumatoid arthritis | - ICD-8: 712,10; 712,20; 712,38; 712,39 - ICD-9: 714A; 714B; 714C; 714W; 719D - ICD-10: M05; M06.0; M06.2; M06.3, M06.8; M06.9; M12.3 |
| Multiple sclerosis | - ICD-8: 340,99 - ICD-9: 340 - ICD-10: G35 |
| Ankylosing spondylitis | - ICD-8: 712,40 - ICD-9: 720A - ICD-10: M45 |
| Spondyloarthritis | - ICD8: 720,1 and 720,2 - ICD-9: 720A; 696A; 713B; 099D; 711A; 720B; 720C; 720W - ICD-10: M45; M08.1; L40.5; M07.0-3; M07.4-5; M07.6; M09.1; M02.0-2; M02.3; M02.8-9; M46.0; M46.1; M46.8; M46.9 |
| Sarcoidosis | - ICD-8: 135 - ICD-9: 135 - ICD-10: ICD-10: D86; G53.2; M63.3 |
| Celiac disease | - ICD-8: 579,0 - ICD-9: 579A - ICD-10: K90.0 |

Abbreviations: ICD, International Classification of Disease.

^1^ Definition of autoimmune disease is based on a previous publication in the same data source with additionally considering celiac disease (exposure in Yuan et al.): Yuan, S. et al. Older age of celiac disease diagnosis and risk of autoimmune disease: A nationwide matched case-control study. J Autoimmun 143, (2024).^5^

^2^ We considered ICD-9 codes between the start of the study period (1992) until 1996, and ICD-10 from 1997 until the end of the study period (2023). Of note, ICD-8 codes (codes used before 1987 in Sweden) are listed in this table, because these codes were – in addition to ICD-9 and ICD-10 codes – considered when defining prior maternal autoimmune disease (covariate).

**Table S5:** Definitions of covariates and baseline characteristic variables (as previously described^1–3^).

| OFFSPRING BASELINE CHARACTERISTICS AND COVARIATES | | | |
| --- | --- | --- | --- |
| Covariate or baseline characteristic variable | **Categories** | **Data source/s** | **Codes/definitions** |
| Female sex | Male  Female | *Total Population Register* | - |
| Calendar year of delivery (i.e., calendar year of date of birth) | 1990-1999  2000-2010  2011-2017 | *MBR* | - |
| Gestational age at birth [weeks] | <37 weeks  <32 weeks  Missing | *MBR* | - |
| Preterm birth | yes/no | *MBR* | <37 gestational weeks  Variables in *MBR:* Induced cesarean section, planned cesarean section: ICD-10: O61; ICD-9: 659B. Exclusion of women with premature rupture of the membranes (ICD-10: O42, ICD-9: 658B)  Variables in *MBR: Spontaneous preterm birth;* premature rupture of the membranes (ICD-10: O42, ICD-9: 658B) |
| Birth weight [g] | Low  Normal  High  Missing | *MBR* | Low: <2,500  Normal: 2,500 to <4,000  High: ≥4,000  Missing |
| Small for gestational age (SGA) | yes/no | *MBR* | Birth weight <10^th^ percentile below the sex specific mean for gestational age according to the Swedish reference curve. |
| Cesarean section | yes/no | *MBR* | Variable in *MBR:* Cesarean section |
| MATERNAL BASELINE CHARACTERISTICS AND COVARIATES | | | |
| Covariate or baseline characteristic variable | **Categories** | **Data source/s** | **Codes/definitions** |
| Age at delivery [years] | <25  25-35  ≥35 | *MBR* | - |
| Country of birth | Nordic  Other  Missing | *Total Population Register* | ~~-~~ |
| Parity | Nulliparous (0 pregnancies prior to current pregnancy)  Multiparous (≥1 pregnancies prior to current pregnancy) | *MBR* | - |
| Level of education [years] | ≤9  10-12  ≥13 years  Missing | *LISA* | - |
| BMI in early pregnancy [kg/m^2^] | <18.5  18.5 to <25  25 to <30  ≥30 | *MBR* | - |
| Smoking in early pregnancy | yes/no | *MBR* | - |
| Autoimmune disease | yes/no | *NPR* | Any time prior to delivery: See ICD-8/ICD-9/ICD-10 codes in Supplementary Table about outcome definition (i.e., autoimmune disease). |
| Any diabetes (pre-existing type 1 or 2 diabetes or gestational diabetes) | yes/no | *MBR* | Type 1 or 2 diabetes (within 5 years prior to delivery): ICD-10: E10, E11; ICD-9: 250; ICD-8: 250  Gestational diabetes (any time prior to delivery): ICD-10: O24.4; ICD-9: 648W |
| Any hypertension (pre-existing hypertension or gestational hypertension) | yes/no | *MBR* | Hypertension (within 5 years prior to delivery): ICD-10: I10-I16, I13-P; ICD-9: 401, 402, 403, 404, 405; ICD-8: 400-404  Gestational hypertension (any time prior to delivery): ICD-10: O13; ICD-9: 760A |
| Dyslipidemia | yes/no | *NPR* and *PDR* | Within 5 years prior to delivery: ICD-10: E78; ICD-9: 272; ICD-8: 272; ATC: C10AA, C10BA, C10BX, C10AB, C10AC |
| Pre-eclampsia | yes/no | *MBR* and *NPR* | Any time prior to delivery: ICD-10: O14-O15; ICD-9: 642E, 642F, 642G, 642H |

Abbreviations: *MBR*, *Medical Birth Register*; *LISA*, Swedish Longitudinal Integrated Database for Health Insurance and Labour Market Studies (*Longitudinell Integrationsdatabas för Sjukförsäkrings- och Arbetsmarknadsstudier*); BMI, body mass index; ICD, International Classification of Diseases; *NPR*, *National Patient Register*; *PDR*, *Prescribed Drug Register*; ATC, Anatomical Therapeutic Chemical; *NPR*, *National Patient Register.*

### **Table S6:** Baseline characteristics of I. offspring of mothers with simple steatosis alone versus reference offspring of mothers without MASLD, as well as baseline characteristics of II. offspring of mothers with severe MASLD versus matched reference offspring of mothers without MASLD.

|  | 1. Simple steatosis | | 1. Severe MASLD* | |
| --- | --- | --- | --- | --- |
|  | Offspring of mothers with MASLD | Reference offspring | Offspring of mothers with MASLD | Reference offspring |
| **Offspring, n** | 175 | 833 | 64 | 298 |
| **Unique mothers, n** | 117 | 831 | 44 | 298 |
| **Years of follow-up** |  |  |  |  |
| Median [IQR] | 19.8 [14.6, 25.2] | 19.7 [14.5, 25.3] | 14.8 [10.8, 17.8] | 14.7 [10.7, 19.9] |
| <10 | 18 (10.3) | 98 (11.8) | 13 (20.3) | 59 (19.8) |
| 10 to <20 | 71 (40.6) | 337 (40.5) | 38 (59.4) | 166 (55.7) |
| ≥20 | 86 (49.1) | 398 (47.8) | 13 (20.3) | 73 (24.5) |
| **OFFSPRING CHARACTERISTICS** |  |  |  |  |
| **Female sex** | 78 (44.8) | 382 (45.9) | 30 (46.9) | 146 (49.0) |
| **Calendar year of date of birth** |  |  |  |  |
| 1992-1999 | 55 (31.4) | 265 (31.8) | 9 (14.1) | 43 (14.4) |
| 2000-2010 | 89 (50.9) | 426 (51.1) | 36 (56.2) | 169 (56.7) |
| 2011-2017 | 31 (17.7) | 142 (17.0) | 19 (29.7) | 86 (28.9) |
| **Gestational age at birth [days], median** **[IQR]** | 274.0 [264.5, 283.0] | 280.0 [273.0, 286.0] | 275.0 [264.0, 283.0] | 282.0 [275.0, 288.0] |
| **Preterm birth (<37 weeks)** | 31 (17.7) | 38 (4.6) | 9 (14.1) | 14 (4.7) |
| **Fetal growth** |  |  |  |  |
| **Birth weight [g]** |  |  |  |  |
| Median [IQR] | 3475 [2963, 3893] | 3595 [3250, 3930] | 3505 [3074, 3903] | 3610 [3235, 3910] |
| Low (<2,500) | 20 (11.4) | 25 (3.0) | 6 (9.4) | 13 (4.4) |
| Normal (2,500 to <4,000) | 121 (69.1) | 630 (75.6) | 45 (70.3) | 223 (74.8) |
| High (≥4,000) | 33 (18.9) | 175 (21.0) | 13 (20.3) | 61 (20.5) |
| Missing | 1 (0.6) | 3 (0.4) | 0 (0.0) | 1 (0.3) |
| **Small for gestational age (SGA)** | 24 (13.8) | 66 (8.0) | 11 (17.2) | 31 (10.4) |
| **Cesarean section** | 56 (32.0) | 129 (15.5) | 21 (32.8) | 52 (17.4) |
| **MATERNAL CHARACTERISTICS** |  |  |  |  |
| **Maternal age at delivery [years]** |  |  |  |  |
| Median [IQR] | 32.0 [28.0, 36.0] | 32.0 [28.0, 36.0] | 30.0 [27.0, 35.0] | 30.0 [27.0, 35.0] |
| 15 to <25 | 13 (7.4) | 61 (7.3) | 13 (20.3) | 62 (20.8) |
| 25 to <35 | 102 (58.3) | 484 (58.1) | 32 (50.0) | 153 (51.3) |
| 35 to 44 | 60 (34.3) | 288 (34.6) | 19 (29.7) | 83 (27.9) |
| **Year of first maternal MASLD diagnosis (index liver biopsy)** |  |  |  |  |
| Up until 1999 | 128 (73.1) | - | 33 (51.6) | - |
| 2000-2010 | 43 (24.6) | - | 28 (43.8) | - |
| 2011-2017 | 4 (2.3) | - | 3 (4.7) | - |
| **Disease duration (time between first MASLD diagnosis and delivery [years]** |  |  |  |  |
| Median [IQR] | 5.8 [3.1, 9.9] | - | 5.3 [3.2, 9.9] | - |
| <5 | 75 (42.9) | - | 27 (42.2) | - |
| 5 to <10 | 58 (33.1) | - | 22 (34.4) | - |
| ≥10 | 42 (24.0) | - | 15 (23.4) | - |
| **Maternal country of birth** |  |  |  |  |
| Nordic | 153 (87.4) | 696 (83.6) | 50 (78.1) | 240 (80.5) |
| Other | 22 (12.6) | 137 (16.4) | 14 (21.9) | 58 (19.5) |
| **Civil status of the mother** |  |  |  |  |
| Living with a partner | 150 (85.7) | 754 (90.5) | 57 (89.1) | 264 (88.6) |
| Not living with a partner | 9 (5.1) | 14 (1.7) | 2 (3.1) | 7 (2.3) |
| Missing | 16 (9.1) | 65 (7.8) | 5 (7.8) | 27 (9.1) |
| **Education** |  |  |  |  |
| Compulsory school (≤9 years) | 26 (14.9) | 83 (10.0) | 10 (15.6) | 33 (11.1) |
| Upper secondary school (10-12 years) | 106 (60.6) | 369 (44.3) | 34 (53.1) | 116 (38.9) |
| College or university (≥13 years) | 43 (24.6) | 366 (43.9) | 20 (31.2) | 142 (47.7) |
| Missing | 0 (0.0) | 15 (1.8) | 0 (0.0) | 7 (2.3) |
| **Parity: multiparous** | 113 (64.6) | 538 (64.6) | 34 (53.1) | 157 (52.7) |
| **BMI in early pregnancy [kg/m²]** |  |  |  |  |
| Median [IQR] | 28.9 [25.0, 33.3] | 24.0 [21.4, 26.8] | 28.0 [25.0, 32.4] | 23.7 [21.6, 27.2] |
| <18.5 | 0 (0.0) | 18 (2.2) | 0 (0.0) | 5 (1.7) |
| 18.5 to <25 | 40 (22.9) | 429 (51.5) | 15 (23.4) | 156 (52.3) |
| 25 to <30 | 49 (28.0) | 202 (24.2) | 23 (35.9) | 69 (23.2) |
| ≥30 | 70 (40.0) | 84 (10.1) | 22 (34.4) | 32 (10.7) |
| Missing | 16 (9.1) | 100 (12.0) | 4 (6.2) | 36 (12.1) |
| **Smoking in early pregnancy** |  |  |  |  |
| Yes | 33 (18.9) | 94 (11.3) | 8 (12.5) | 20 (6.7) |
| No | 135 (77.1) | 695 (83.4) | 52 (81.2) | 264 (88.6) |
| Missing | 7 (4.0) | 44 (5.3) | 4 (6.2) | 14 (4.7) |
| **Prior comorbidities and conditions** |  |  |  |  |
| Autoimmune disease | 35 (20.0) | 22 (2.6) | 9 (14.1) | 12 (4.0) |
| Diabetes^1^ | 12 (6.9) | 9 (1.1) | 13 (20.3) | 1 (0.3) |
| Hypertension^2^ | 9 (5.1) | 4 (0.5) | 3 (4.7) | 2 (0.7) |
| Dyslipidemia | 3 (1.7) | 1 (0.1) | 1 (1.6) | 1 (0.3) |
| Pre-eclampsia | 15 (8.6) | 19 (2.3) | 4 (6.2) | 16 (5.4) |

Values are n (%), unless otherwise indicated.

Abbreviations: MASLD, metabolic dysfunction-associated steatotic liver disease; n, number; IQR, interquartile range; BMI, body mass index.

Of note, a similar table has been presented in our previous studies.^1,2^

^1^ Defined as simple steatosis plus MASH without fibrosis, noncirrhotic fibrosis, or cirrhosis.

^2^ Diabetes type 1, diabetes type 2, or gestational diabetes.

^+^Including gestational hypertension.

### **Table S7:** Characteristics of autoimmune disease defined by ≥1 diagnosis code for autoimmune disease or ≥1 prescribed medication uniquely indicated for autoimmune disease during follow-up (sensitivity analysis).

|  | Overall | Offspring of mothers with MASLD | Reference  Offspring |
| --- | --- | --- | --- |
| Number of offspring with autoimmune disease (%) | 69 (5.0) | 22 (9.2) | 47 (1.9) |
| Age at autoimmune disease [years] |  |  |  |
| Median [IQR] | 12.9 [4.7, 19.5] | 17.1 [7.6, 22.4] | 9.3 [4.7, 18.4] |
| <10 | 31 (44.9) | 7 (31.8) | 24 (51.1) |
| 10 to <20 | 21 (30.4) | 8 (36.4) | 13 (27.7) |
| ≥20 | 17 (24.6) | 7 (31.8) | 10 (21.3) |
| Type of autoimmune disease (in descending order^1^) |  |  |  |
| Celiac disease | 15 (21.7) | 2 (9.1) | 13 (27.7) |
| Type 1 diabetes | 14 (20.3) | 3 (13.6) | 11 (23.4) |
| Psoriasis | 12 (17.4) | 5 (22.7) | 7 (14.9) |
| Autoimmune thyroiditis  (Hashimoto’s thyroiditis) | 11 (15.9) | 4 (18.2) | 7 (14.9) |
| Inflammatory bowel disease | 6 (8.7) | 3 (13.6) | 3 (6.4) |
| Alopecia areata | 3 (4.3) | NR | NR |
| Grave’s disease | NR | NR | NR |
| Spondyloarthritis | NR | NR | NR |
| Systemic/cutaneous lupus erythematosus | NR | NR | NR |
| Rheumatoid arthritis | NR | NR | NR |
| Vitiligo | NR | NR | NR |

Values are n (%), unless otherwise indicated.

Abbreviations: MASLD, metabolic dysfunction-associated steatotic liver disease; n, number; IQR, interquartile range, NR, not reported (data privacy concerns).

^1^ Table only shows specific autoimmune diseases for which two or more births with the diagnosis respectively medication were found in both groups (protection of data privacy).

**Table S8:** Cousin-controlled analysis: Baseline characteristics of mothers with MASLD, their sisters (without known MASLD), and their offspring.

|  | **Offspring of mothers with MASLD** | **Offspring of sisters of mothers with MASLD (i.e., first cousins)** |
| --- | --- | --- |
| **Offspring, n** | 78 | 125 |
| **Unique mothers, n** | 45 | 59 |
| **Years of follow-up** |  |  |
| Median [IQR] | 17.1 [12.5, 22.6] | 20.8 [15.4, 25.5] |
| <10 | 11 (14.1) | 5 (4.0) |
| 10 to <20 | 40 (51.3) | 56 (44.8) |
| ≥20 | 27 (34.6) | 64 (51.2) |
| **OFFSPRING CHARACTERISTICS** |  |  |
| **Female sex** | 35 (44.9) | 59 (47.2) |
| **Calendar year of date of birth** |  |  |
| 1992-1999 | 16 (20.5) | 43 (34.4) |
| 2000-2010 | 41 (52.6) | 67 (53.6) |
| 2011-2017 | 21 (26.9) | 15 (12.0) |
| **Gestational age at birth [days], median [IQR]** | 272.5 [260.0, 282.8] | 279.0 [272.0, 287.0] |
| **Preterm birth (<37 weeks)** | 16 (20.5) | 4 (3.2) |
| **Fetal growth** |  |  |
| **Birth weight [g]** |  |  |
| Median [IQR] | 3505 [2960, 3883] | 3646 [3400, 3960] |
| Low (<2,500) | 10 (12.8) | 3 (2.4) |
| Normal (2,500 to <4,000) | 54 (69.2) | 96 (76.8) |
| High (≥4,000) | 14 (17.9) | 26 (20.8) |
| **Small for gestational age (SGA)** | 8 (10.3) | 8 (6.4) |
| **Cesarean section** | 32 (41.0) | 24 (19.2) |
| **MATERNAL CHARACTERISTICS** |  |  |
| **Maternal age at delivery [years]** |  |  |
| Median [IQR] | 30.0 [27.0, 35.0] | 28.0 [25.0, 32.0] |
| 15 to <25 | 8 (10.3) | 27 (21.6) |
| 25 to <35 | 48 (61.5) | 79 (63.2) |
| 35 to 44 | 22 (28.2) | 19 (15.2) |
| **Liver histology of maternal MASLD** |  |  |
| Simple steatosis | 55 (70.5) | - |
| MASH without fibrosis | 9 (11.5) | - |
| Noncirrhotic fibrosis | 10 (12.8) | - |
| Cirrhosis | 4 (5.1) | - |
| **Year of first maternal MASLD diagnosis (index liver biopsy)** |  |  |
| Up until 1999 | 49 (62.8) | - |
| 2000-2010 | 27 (34.6) | - |
| 2011-2017 | 2 (2.6) | - |
| **Disease duration (time between first MASLD diagnosis and delivery [years]** |  |  |
| Median [IQR] | 5.7 [3.0, 9.2] | - |
| <5 | 31 (39.7) | - |
| 5 to <10 | 31 (39.7) | - |
| ≥10 | 16 (20.5) | - |
| **Maternal country of birth** |  |  |
| Nordic | 75 (96.2) | 125 (100.0) |
| Other | 3 (3.8) | 0 (0.0) |
| **Civil status of the mother** |  |  |
| Living with a partner | 65 (83.3) | 116 (92.8) |
| Not living with a partner | 5 (6.4) | 3 (2.4) |
| Missing | 8 (10.3) | 6 (4.8) |
| **Education** |  |  |
| Compulsory school (≤9 years) | 7 (9.0) | 22 (17.6) |
| Upper secondary school (10-12 years) | 48 (61.5) | 81 (64.8) |
| College or university (≥13 years) | 23 (29.5) | 22 (17.6) |
| **Parity: multiparous** | 48 (61.5) | 79 (63.2) |
| **BMI in early pregnancy [kg/m²]** |  |  |
| Median [IQR] | 29.3 [25.7, 35.4] | 25.0 [22.2, 28.7] |
| <18.5 | 0 (0.0) | 0 (0.0) |
| 18.5 to <25 | 15 (19.2) | 55 (44.0) |
| 25 to <30 | 26 (33.3) | 33 (26.4) |
| ≥30 | 30 (38.5) | 23 (18.4) |
| Missing | 7 (9.0) | 14 (11.2) |
| **Smoking in early pregnancy** |  |  |
| Yes | 9 (11.5) | 13 (10.4) |
| No | 66 (84.6) | 106 (84.8) |
| Missing | 3 (3.8) | 6 (4.8) |
| **Prior comorbidities and conditions** |  |  |
| Autoimmune disease | 9 (11.5) | 6 (4.8) |
| Diabetes^1^ | 8 (10.3) | 4 (3.2) |
| Hypertension^2^ | 2 (2.6) | 1 (0.8) |
| Dyslipidemia | 1 (1.3) | 0 (0.0) |
| Pre-eclampsia | 11 (14.1) | 4 (3.2) |

Values are n (%), unless otherwise indicated.

Abbreviations: MASLD, metabolic dysfunction-associated steatotic liver disease; n, number; IQR, interquartile range; MASH, metabolic dysfunction-associated steatohepatitis; BMI, body mass index.

Of note, a similar table has been presented in our previous studies.^1,2^

^1^ Diabetes type 1, diabetes type 2, or gestational diabetes.

^2^ Including gestational hypertension.

**Table S9:** Sensitivity analyses: Autoimmune disease among offspring exposed in utero to maternal MASLD and reference offspring.

|  | **N** | **Events** | **py** | **IR per 1000 py (95% CI)** | **Crude HR (95% CI)**  Model 1* | **Adjusted HR (95% CI)**  Model 2** |
| --- | --- | --- | --- | --- | --- | --- |
| **Confirmed autoimmune disease (≥2 diagnoses)** |  |  |  |  |  |  |
| Reference offspring | 1131 | 36 | 20843 | 1.7 (1.2-2.4) | 1 (Reference) | 1 (Reference) |
| Offspring of mothers with MASLD | 239 | 9 | 4450 | 2.0 (0.9-3.8) | 1.17 (0.56-2.43) | 0.71 (0.28-1.78) |
| **Autoimmune disease: ≥1 diagnosis or ≥1 medication** |  |  |  |  |  |  |
| Reference offspring | 1131 | 47 | 20751 | 2.3 (1.7-3.0) | 1 (Reference) | 1 (Reference) |
| Offspring of mothers with MASLD | 239 | 22 | 4351 | 5.1 (3.2-7.7) | 2.23 (1.34-3.70) | 1.65 (0.88-3.11) |
| **(Maternal) MASLD diagnosis before start of pregnancy and no ICP during pregnancy of interest** |  |  |  |  |  |  |
| Reference offspring | 1103 | 39 | 20272 | 1.9 (1.4-2.6) | 1 (Reference) | 1 (Reference) |
| Offspring of mothers with MASLD | 233 | 15 | 4311 | 3.5 (1.9-5.7) | 1.81 (1.00-3.27) | 1.20 (0.57-2.52) |
| **Normal birth weight for gestational age and term born and not born via cesarean section** |  |  |  |  |  |  |
| Reference offspring | 470 | 23 | 9318 | 2.5 (1.6-3.7) | 1 (Reference) | 1 (Reference) |
| Offspring of mothers with MASLD | 127 | 9 | 2461 | 3.7 (1.7-6.9) | 1.49 (0.69-3.23) | 1.15 (0.43-3.07) |

Abbreviations: py, person-years; IR, incidence rate; CI, confidence interval; HR, hazard ratio; MASLD, metabolic dysfunction-associated steatotic liver disease; ICP, intrahepatic cholestasis of pregnancy.

*Model 1: conditioned on matching set (maternal age at delivery, calendar year of delivery, and parity).

**Model 2: conditioned on matching set and further adjusted for the offspring’s sex and the following maternal factors: level of education, any metabolic disorder recorded any time prior to delivery of the offspring of interest (any diabetes, any hypertension, obesity [BMI in early pregnancy ≥30 kg/m^2^], dyslipidemia, pre-eclampsia), any autoimmune disease except type 1 diabetes recorded any time prior to delivery of the offspring of interest, and smoking status in early pregnancy.

**Table S10:** Characteristics of the outcome when autoimmune disease was confirmed by a second (confirmatory) diagnosis during follow-up.

|  | Overall | Offspring of mothers with MASLD | Reference  offspring |
| --- | --- | --- | --- |
| Autoimmune disease during follow-up, n | 45 (3.3) | 9 (3.8) | 36 (3.2) |
| Age at autoimmune disease [years] |  |  |  |
| Median [IQR] | 9.5 [6.2, 16.2] | 17.3 [9.5, 17.7] | 9.2 [4.6, 15.4] |
| <10 | 23 (51.1) | 3 (33.3) | 20 (55.6) |
| 10 to <20 | 15 (33.3) | 4 (44.4) | 11 (30.6) |
| ≥20 | 7 (15.6) | 2 (22.2) | 5 (13.9) |
| Type of autoimmune disease (in descending order^1^) |  |  |  |
| Type 1 diabetes | 15 (33.3) | 3 (33.3) | 12 (33.3) |
| Celiac disease | 12 (26.7) | 0 (0.0) | 12 (33.3) |
| Inflammatory bowel disease | 7 (15.6) | 3 (33.3) | 4 (11.1) |
| Psoriasis | 4 (8.9) | 0 (0.0) | 4 (11.1) |
| Grave’s disease | 3 (6.7) | NR | NR |
| Systemic/cutaneous lupus erythematosus | NR | NR | NR |
| Alopecia areata | NR | NR | NR |
| Rheumatoid arthritis | NR | NR | NR |

Values are n (%), unless otherwise indicated.

Abbreviations: MASLD, metabolic dysfunction-associated steatotic liver disease; n, number; IQR, interquartile range, NR, not reported (data privacy concerns).

^1^ Table only shows specific autoimmune diseases for which two or more births with the diagnosis were found in both groups (protection of data privacy).


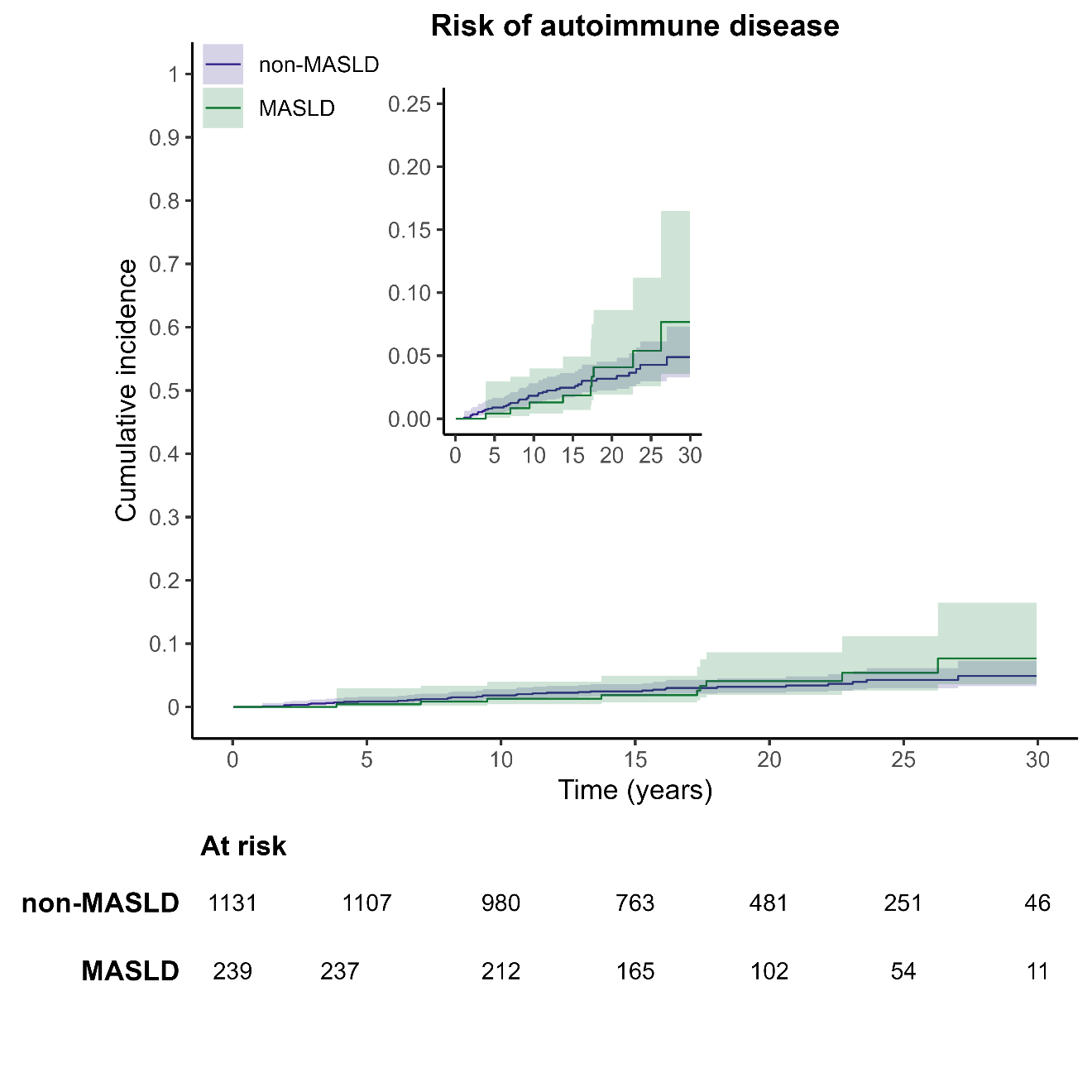


**Figure S2:** Crude cumulative incidence curves for confirmed autoimmune disease (≥2 diagnosis codes) in offspring born to mothers with MASLD vs. reference offspring of mothers without known MASLD.


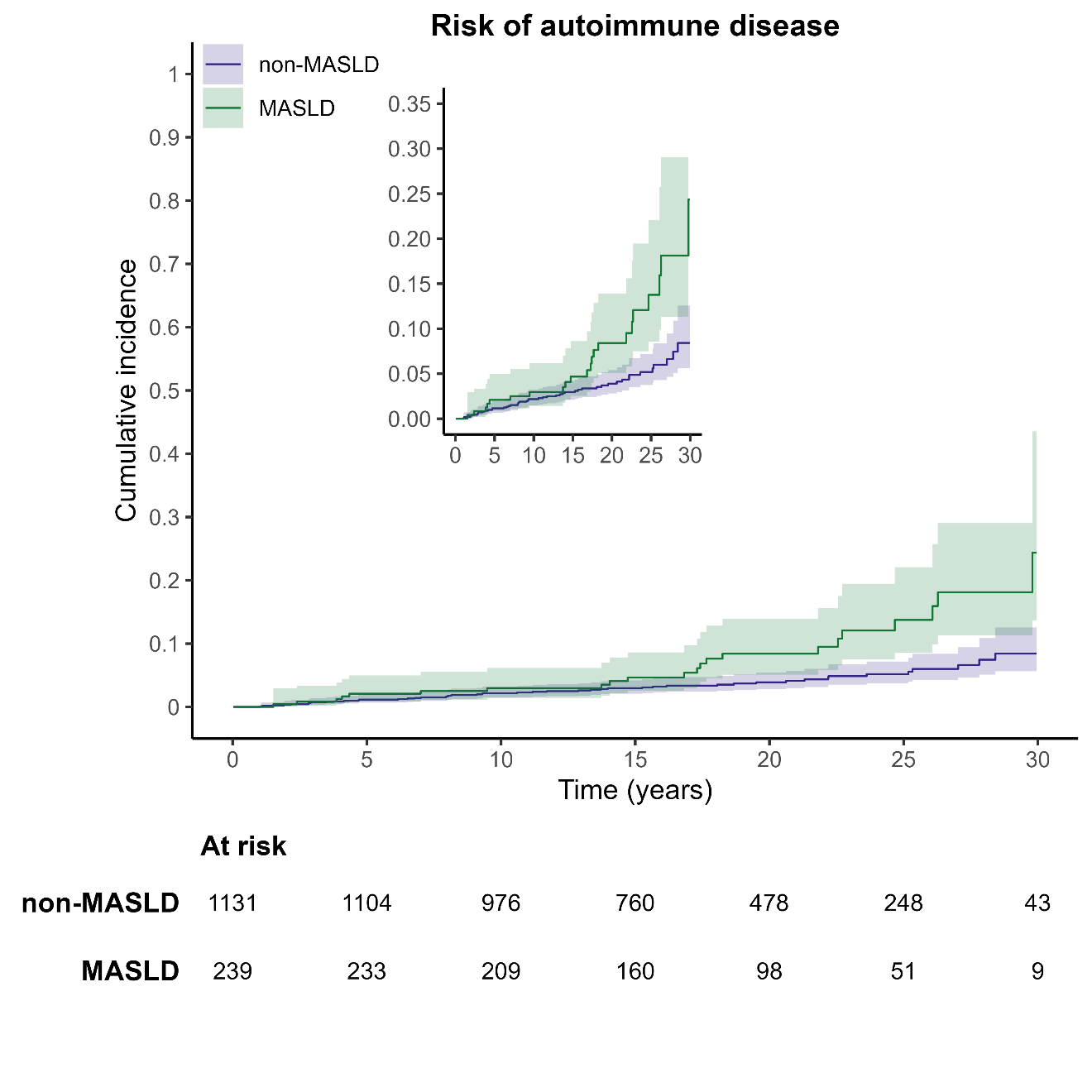


**Figure S3:** Crude cumulative incidence curves for autoimmune disease (≥1 diagnosis or ≥1 medication) in offspring born to mothers with MASLD vs. reference offspring of mothers without known MASLD.

### **References (Supplementary Material)**

1. Marxer CA, Ebrahimi F, Bergman D, Sun J, Hagström H, Thuresson M, Stephansson O, Ludvigsson JF. Mortality and Cancer in Offspring of Mothers With Biopsy-Proven MASLD During Pregnancy: A Nationwide Cohort Study. *Liver International*. 2025;45:70174. doi:10.1111/liv.70174

2. Marxer CA, Ebrahimi F, Bergman D, Sun J, Hagström H, Thuresson M, Stephansson O, Ludvigsson JF. Higher Risk of Serious Infection in Offspring of Mothers With Biopsy-Proven MASLD: A Nationwide Cohort Study. *United European Gastroenterol J*. 2026;14(1):e70163. doi:10.1002/UEG2.70163

3. Marxer CA, Ebrahimi F, Bergman D, Sun J, Hagström H, Thuresson M, Stephansson O, Ludvigsson JF. Adverse pregnancy and birth outcomes in women with biopsy-proven MASLD: a nationwide cohort study. *EClinicalMedicine*. Published online May 9, 2025:103238. doi:10.1016/J.ECLINM.2025.103238

4. Svensk Förening för Patologi. Svensk Förening för Klinisk Cytologi. Accessed April 4, 2019. https://www.svfp.se/foreningar/uploads/L15178/kvast/lever/Leverbiopsier2019.pdf

5. Yuan S, Leffler D, Lebwohl B, Green PHR, Larsson SC, Söderling J, Sun J, Ludvigsson JF. Older age of celiac disease diagnosis and risk of autoimmune disease: A nationwide matched case-control study. *J Autoimmun*. 2024;143. doi:10.1016/J.JAUT.2024.103170
